# Complete Mitochondrial Genome Sequencing of Asian Glass Lizards (Anguidae: *Dopasia*): Comparative Analysis With Limbless Anguids and New Insights Into the Adaptive Evolution of Protein‐Coding Genes

**DOI:** 10.1002/ece3.72811

**Published:** 2025-12-25

**Authors:** Bo Cai, Chengguanyu Liu, Gaus Shang, Yingyong Wang, Sikan Chen, Dong Liang, Wei Zeng, Shichao Zheng, Xianguang Guo

**Affiliations:** ^1^ China‐Croatia Belt and Road Joint Laboratory on Biodiversity and Ecosystem Services, Chengdu Institute of Biology Chinese Academy of Sciences Chengdu China; ^2^ Mountain Ecological Restoration and Biodiversity Conservation Key Laboratory of Sichuan Province Chengdu Institute of Biology, Chinese Academy of Sciences Chengdu China; ^3^ University of Chinese Academy of Sciences Beijing China; ^4^ Department of Biotechnology Ming Chuan University Taoyuan China; ^5^ State Key Laboratory of Biocontrol/The Museum of Biology, School of Life Sciences Sun Yat‐sen University Guangzhou Guangdong China; ^6^ Guizhou Institute of Forestry Inventory and Planning Guiyang Guizhou China; ^7^ Sichuan Mt. Emei Forest Ecosystem National Observation and Research Station Emeishan Sichuan China; ^8^ Mount Emei Scenic Area Management Committee Emeishan Sichuan China; ^9^ Fujian Research and Monitoring Center of Wuyi Mountain National Park Wuyishan Fujian China

**Keywords:** Anguidae, *Dopasia*, evolution, mitochondrial genome, phylogeny, positive selection

## Abstract

The mitochondrial genome (mitogenome) plays a critical role in energy production and is frequently subject to positive selection due to its impact on adaptive evolution. In this study, we investigated positive selection pressures in the mitogenomes of *Dopasia* (Anguidae), a genus that occupies diverse habitats. Using next‐generation sequencing, we determined the complete mitogenomes of eight individuals (six *D. gracilis* and two *D. harti* ) and compared them with 28 published mitogenomes of the Anguidae family from GenBank. Although purifying selection is the predominant evolutionary force, six of the 13 protein‐coding genes showed signatures of positive selection. Notably, amino acid preferences at these sites correlated with the geographic distributions of the species, suggesting the involvement of the mitogenome in environmental adaptation. We also mapped these sites onto three‐dimensional protein structures and transmembrane domains. Phylogenetic analyses resolved *Dopasia*'s relationship with other Anguidae species, but challenged the monophyly of *D. hainanensis* within the genus. Our findings highlight the potential role of the mitogenome in ecological adaptation and underscore the need for a broader sampling of Anguidae mitogenomes to clarify evolutionary mechanisms.

## Graphical abstract

This study analyzed mitochondrial genomes from eight individuals of two *Dopasia* species and 28 other Anguidae sequences to investigate selection patterns in protein‐coding genes. Positive selection was detected in six of the 13 mitochondrial genes, with amino acid preferences correlating with geographic distribution, suggesting a role in environmental adaptation. The findings highlight the importance of mitochondrial genomic variation in adaptive evolution and raise questions about the phylogeny and taxonomy within the family Anguidae.

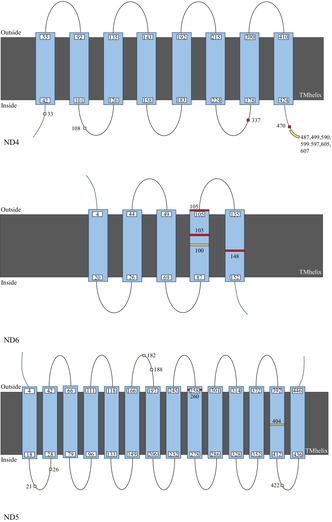

## 1 Introduction

The vertebrate mitochondrial genome (mitogenome) typically consists of 37 genes: 13 protein‐coding genes (PCGs), 22 transfer RNAs (tRNAs), and two ribosomal RNAs (rRNAs). These genes are all essential for oxidative phosphorylation (OXPHOS), the primary pathway of ATP production (Das 2006). The PCGs encode subunits of the main OXPHOS complexes, such as the NADH dehydrogenases (ND1‐6 and ND4L), cytochrome c oxidases (COI‐III), and cytochrome *b* (Cyt *b*), as well as the ATP synthase (ATP6 and ATP8). Due to their pivotal role in energy metabolism, mitogenomes are under significant evolutionary pressure, striking a balance between purifying selection, which maintains core functions, and positive selection, which drives adaptation to ecological niches (Embley and Martin 2006).

Discrepancies between an organism's energy requirements and its environment can impose genetic constraints on oxidative phosphorylation capacity (Embley and Martin 2006). For instance, limbless lizards in the Anguimorpha infraorder exhibit positively selected sites in cytochrome *b* (Cyt *b*), NADH dehydrogenase 2 (ND2), NADH dehydrogenase 3 (ND3), NADH dehydrogenase 5 (ND5), and NADH dehydrogenase 6 (ND6), which are likely to reflect adaptations to energy demands associated with locomotion (Zhan et al. 2024). Such findings highlight the potential role of the mitogenome in speciation and environmental adaptation (Avise et al. 1979; Avise 1986).

Ecological opportunities, environmental conditions that favor diversification, can shape the evolution of mitochondrial genomes by selecting for variants that optimize energy production under local constraints (Wellborn and Langerhans 2015). Although purifying selection is dominant due to functional constraints (Ballard and Whitlock 2004), positive selection has been observed in vertebrate mitochondrial genomes, particularly in lineages that colonize new habitats (Sahoo et al. 2023; Zhang et al. 2021). For example, the COXI gene in *Dopasia* and other limbless anguimorphs exhibits adaptive signals associated with habitat‐specific energy requirements (Zhan et al. 2024). However, the extent of positive selection in understudied lineages such as *Dopasia* remains unclear.

The genus *Dopasia* (Anguidae), distributed across the Indochina subregion (Nguyen et al. 2011), has not been well characterized genetically. As of October 2024, only 28 complete Anguidae mitogenomes were available in GenBank, with phylogenetic analyses recovering *Dopasia* as sister to the North American *Ophisaurus* (Zhan et al. 2024). While three species are recognized in China— *D. harti* , *D. hainanensis* , and *D. gracilis* (Cai et al. 2015)—recent studies suggest that *D. hainanensis* and the Taiwanese *Ophisaurus formosensis* may both be synonyms of *D. harti* , based on low genetic divergence (< 8%) in Cyt *b* and minimal morphological differentiation (Li et al. 2024). These conclusions remain provisional, highlighting the need for expanded genomic sampling to resolve persistent taxonomic uncertainties.

Here, we sequenced eight complete mitogenomes (six *D. gracilis* and two *D. harti* ) and analyzed them alongside 28 published Anguidae mitogenomes to: (i) reconstruct the phylogeny of *Dopasia* and assess the monophyly of *D. hainanensis* , (ii) identify signatures of positive selection in mitochondrial PCGs and map these sites to protein structures, (iii) examine the correlation between amino acid substitutions and geographic distribution, and (iv) evaluate the role of mitogenome evolution in ecological adaptation. Our study provides new light on the evolutionary mechanisms shaping *Dopasia* diversification and highlights the importance of mitogenomes in adapting to heterogeneous environments.

## 2 Materials and Methods

### 2.1 Sample Collection and DNA Extraction

**FIGURE 1:**
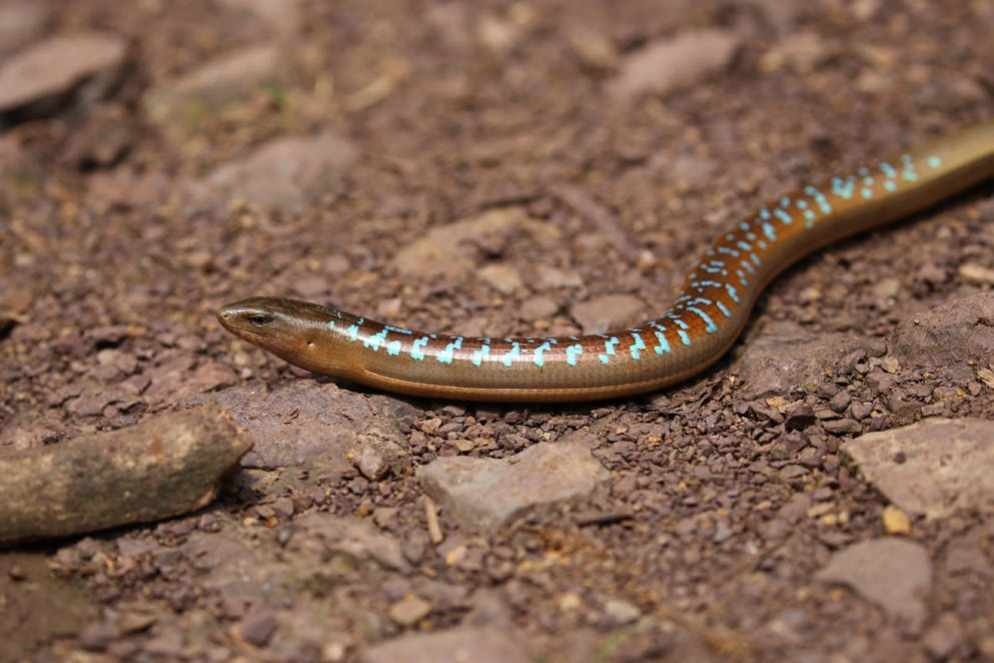
A male Hart's glass lizard ( *Dopasia harti* ) from Mt. Emei, Sichuan, China. Photo by Bo Cai.

We collected eight tissue samples from *Dopasia* specimens across China (Table 1). A photograph of one of the study organisms, *D. harti* , is present in Figure 1. Animals were humanely euthanized with intraperitoneal injection of pentobarbital sodium (overdose protocol), following ethical guidelines approved by the Animal Care and Use Committee of the Chengdu Institute of Biology (CIB; approval number CIB‐20121220A). Liver tissues and voucher specimens were preserved in 95% ethanol and stored at CIB, Chinese Academy of Sciences. Total genomic DNA was extracted from liver tissue using a high‐salt extraction protocol (Aljanabi and Martinez 1997).

**TABLE 1:** Taxon information and GenBank accession numbers for Anguidae and two outgroup species.

Taxon	Family	Accession number	Length (bp)	Country	Locality	References
*Dopasia*	Anguidae					
*Dopasia gracilis* B5008	Anguidae		17,218	China	Tibet	This study
*Dopasia gracilis* G1942	Anguidae		17,241	China	Guangxi	This study
*Dopasia gracilis* MTX	Anguidae		16,823	China	Tibet	This study
*Dopasia gracilis* GZ18001	Anguidae		16,775	China	Guizhou	This study
*Dopasia gracilis* GD04	Anguidae		17,070	China	Fujian	This study
*Dopasia gracilis* S0867	Anguidae		17,272	China	Guangxi	This study
*Dopasia harti* TW	Anguidae		17,069	China	Taiwan	This study
*Dopasia harti* CB	Anguidae		17,000	China	Sichuan	This study
*Dopasia hainanensis*	Anguidae	MN640999	17,000	China	Hainan	Gvoždík et al. (2023)
*Dopasia harti*	Anguidae	KF806482	17,571	China	Anhui	Yan (2015)
*Dopasia harti*	Anguidae	KF279681	17,163	China	Anhui	Pan et al. (2015)
*Dopasia gracilis*	Anguidae	MN661343	17,133	China	Yunnan	Cai, Guo, and Jiang (2020), Cai, Guo, Song, and Chen (2020)
*Dopasia gracilis*	Anguidae	KJ941042	17,788	China	Yunnan	Yan (2015)
*Dopasia gracilis*	Anguidae	KU885977	16,777	China	Tibetan Plateau	Fang (Unpubl. data)
*Dopasia sokolovi*	Anguidae	OP493527	17,137	Vietnam	Bidoup‐Nui Ba	Gvoždík et al. (2023)
*Anguis*	Anguidae					
*Anguis graeca*	Anguidae	OP493517	17,103	Greece	Mornos River	Gvoždík et al. (2023)
*Anguis graeca*	Anguidae	KX236331	19,297	N.A.	N.A.	Strzala (Unpubl. data)
*Anguis fragilis*	Anguidae	OP493518	17,096	Czech	Rep. Zelízy	Gvoždík et al. (2023)
*Anguis fragilis*	Anguidae	OP493519	17,103	Spain	Torla‐Ordesa	Gvoždík et al. (2023)
*Anguis fragilis*	Anguidae	EU443256	17,479	Spain	Vilarmiel Lugo	Albert et al. (2009)
*Anguis fragilis*	Anguidae	MN122840	17,134	N.A.	N.A.	Margaryan (Unpubl. data)
*Anguis colchica*	Anguidae	OP493520	17,383	Czech	Rep. Ostravice	Gvoždík et al. (2023)
*Anguis colchica*	Anguidae	OP493521	17,390	Lithuania	Marcinkony	Gvoždík et al. (2023)
*Anguis colchica*	Anguidae	OP493522	17,420	Iran	Motalla Sara‐ye Lemir	Gvoždík et al. (2023)
*Anguis colchica*	Anguidae	OP493523	17,125	Bulgaria	Sinemore	Gvoždík et al. (2023)
*Anguis colchica*	Anguidae	OP493529	17,168	Georgia	Telavi	Gvoždík et al. (2023)
*Anguis colchica*	Anguidae	KX236330	17,097	Poland	Bieszczady Mts. Zatwarnica village	Strzała, Grochowalska, Najbar, Najbar, and Jablonski (2017)
*Anguis veronensis*	Anguidae	OP493530	17,186	Italy	Roccagnano	Gvoždík et al. (2023)
*Anguis veronensis*	Anguidae	KX236332	17,322	Italy	Bergamo	Strzała, Grochowalska, Najbar, Crottini, et al. (2017)
*Anguis cephallonica*	Anguidae	OP493528	17,224	Greece	Gialova	Gvoždík et al. (2023)
*Anguis cephallonica*	Anguidae	KU052866	17,208	Greece	Peloponnese Peninsula	Strzała et al. (2016)
*Pseudopus*	Anguidae					
*Pseudopus apodus*	Anguidae	OP493524	16,701	Georgia	Dedop'lis Tskaro	Gvoždík et al. (2023)
*Pseudopus apodus*	Anguidae	OP493526	16,901	Albania	Zogaj	Gvoždík et al. (2023)
*Pseudopus apodus*	Anguidae	OP493525	17,104	Israel	Hare Gilboa’	Gvoždík et al. (2023)
*Ophisaurus*	Anguidae					
*Ophisaurus attenuatus*	Anguidae	EU747729	14,628	N.A.	N.A.	Castoe et al. (2008)
*Abronia*	Anguidae					
*Abronia graminea*	Anguidae	AB080273	16,016	N.A.	N.A.	Kumazawa and Nishida (1995)
**Outgroups**						
*Heloderma suspectum*	Helodermatidae	AB167711	16,846	Japan	Nagoya Higashiyama Zoo	Kumazawa (2007)
*Shinisaurus crocodilurus*	Shinisauridae	HQ008865	16,585	N.A.	N.A.	Li et al. (2012)
*Varanus salvator*	Varanidae	AB980995	17,536	Thailand	Nakhon Ratchasima Zoo	Chaiprasertsri et al. (2013)

### 2.2 Library Construction and High‐Throughput Sequencing

DNA sample integrity was verified via 1% agarose gel electrophoresis, and purity was assessed using a NanoDrop 2000 spectrophotometer (Thermo Scientific, Wilmington, DE, USA) and a Qubit 3.0 Flurometer (Invitrogen, Carlsbad, CA, USA). Qualified DNA samples were randomly sheared to ~350 bp fragments. The entire library preparation process was then carried out, including end‐repair, addition of an A base at the 3′ end, ligation of sequencing adapters, purification, and PCR amplification.

Library quality was validated by Qubit 3.0 Fluorometer for preliminary quantification, Bioanalyzer 2100 (Agilent) or TapeStation to confirm insert size, and quantitative PCR (Q‐PCR) (KAPA Library Quant Kit) to measure effective concentration (> 3 nM). Final libraries were sequenced on an Illumina HiSeq X platform (150 bp paired‐end; Beijing Tsingke Biotech Co. Ltd.), generating ~20 Gb raw data per sample.

### 2.3 Sequence Assembly, Annotation, and Analysis

The raw sequencing data from Illumina HiSeq were processed using fastp v0.20.0 (https://github.com/OpenGene/fastp, accessed on 22 March 2024) for quality control. This involved adapter trimming, removal of low‐quality reads (Phred score < Q5), and filtering of sequences containing excessive ambiguous bases. The resulting high‐quality reads were then aligned against the reference *Dopasia harti* mitogenome (GenBank accession KJ941042) to identify and remove redundant or erroneous sequences. We performed de novo assembly using SPAdes v3.10.1 (Bankevich et al. 2012) to reconstruct complete circular mitochondrial genomes, with careful manual inspection of the assembly graphs to ensure accuracy.

For sequence verification, we conducted BLAST searches against the NCBI nucleotide database and performed manual curation using MEGA v7.0.26 (Kumar et al. 2016). Gene annotation involved multiple approaches: protein‐coding genes were verified through ORF prediction and BLASTx comparisons, tRNA genes were identified using tRNAscan‐SE 2.0 (Chan and Lowe 2019) with default parameters, and rRNA genes were annotated by alignment to known Anguidae sequences. The control region was determined by its characteristic position between tRNA‐Phe and tRNA‐Pro genes.

We analyzed various genomic features including nucleotide composition, codon usage, and strand asymmetry. The AT and GC skews were calculated using standard formulas: AT skew = (A − T)/(A + T) and GC skew = (G − C)/(G + C). For visualization, the complete mitogenomes were mapped using OGDRAW v1.3.1 (Greiner et al. 2019). Our final dataset incorporated 38 mitochondrial genomes, including eight newly sequenced specimens and 28 publicly available sequences from GenBank, with full taxonomic details and accession numbers provided in Table 1.

### 2.4 Phylogenetic Analysis

We conducted phylogenetic reconstruction using both Maximum Likelihood (ML) and Bayesian Inference (BI) approaches. All analyses were performed through PhyloSuite v1.2.3 (Zhang et al. 2020; Xiang et al. 2023), which streamlined the workflow from gene partitioning to tree construction. For partitioning strategy and model selection, we employed PartitionFinder v2.1.1 (Lanfear et al. 2017) with the greedy algorithm and corrected Akaike Information Criterion (AICc) (Lanfear et al. 2012). The “all” mode was used for ML analysis, while the “Mrbayes” mode (Ronquist and Huelsenbeck 2003; Ronquist et al. 2012) was selected for BI analysis to determine optimal partitioning schemes and substitution models for each dataset.

For Bayesian analysis, we ran two independent Markov Chain Monte Carlo (MCMC) runs in MrBayes v3.2.7a for 20 million generations, sampling trees every 1000 generations. Convergence was assessed by monitoring the standard deviation of split frequencies between runs, with values below 0.01 indicating satisfactory convergence. After discarding the first 25% of trees as burn‐in, we generated a 50% majority‐rule consensus tree from the remaining trees. Nodes with posterior probabilities (PP) ≥ 0.95 were considered strongly supported (Erixon et al. 2003; Huelsenbeck and Rannala 2004).

Maximum Likelihood analysis was performed using IQ‐TREE v2.2.0 (Nguyen et al. 2015) with an ultrafast bootstrap approximation of 5000 replicates. Branch support was assessed using ultrafast bootstrap values (UFBoot), with nodes scoring ≥ 95% considered well‐supported (Minh et al. 2013). The resulting phylogenetic trees were visualized using ChiPlot v2.6 (Xie et al. 2023), with final graphical adjustments made in Microsoft PowerPoint. The best‐fit substitution models and partitioning schemes for protein‐coding genes are presented in Table 2.

**TABLE 2:** Best‐fit models and partitioning schemes selected by PartitionFinder 2.

Partition names	Length (bp)	Best model
ATP6	693	GTR + I + G
ATP8	165	GTR + I + G
COX1	1548	GTR + I + G
COX2	687	GTR + I + G
CYTB	1134	GTR + I + G
ND1	972	GTR + I + G
ND2	1035	GTR + I + G
ND3	345	GTR + I + G
ND4L	294	GTR + I + G
ND4	1380	GTR + I + G
ND5	1827	GTR + I + G
ND6	534	HKY + I + G

### 2.5 Selection Pressure Analysis

We analyzed selective pressures on protein‐coding genes by calculating nonsynonymous to synonymous substitution rate ratios (*ω* = dN/dS) using PhyloSuite v1.2.3 (Zhang et al. 2020; Xiang et al. 2023), excluding stop codons from all analyses. The CodeML program (PAML v4.10.7) (Xu and Yang 2013; Yang 2007) was employed to test for signatures of selection across the mitochondrial phylogeny. We implemented five evolutionary models on concatenated coding sequences: M0 (single *ω* ratio), M1a (nearly neutral), M2a (positive selection), M7 (beta distribution), and M8 (beta & *ω*). Likelihood ratio tests (LRTs) compared nested models to assess evolutionary patterns, specifically testing M0 versus M1a for selection variability, and M1a versus M2a and M7 versus M8 for positive selection signals. Sites were considered under positive selection if they showed *ω* > 1 in M2a/M8 models with significant LRTs (*p* < 0.05), with posterior probabilities calculated using Bayesian Empirical Bayes (BEB).

Complementary analyses were performed using HyPhy (Kosakovsky Pond et al. 2020; Kosakovsky Pond et al. 2005) through the Datamonkey webserver (Delport et al. 2010; Kosakovsky Pond et al. 2005; Weaver et al. 2018), implementing three distinct approaches: FUBAR (Murrell et al. 2013) (posterior probability > 0.9), MEME (Murrell et al. 2012) (*p* ≤ 0.05), and SLAC (Kosakovsky Pond et al. 2005) (*p* ≤ 0.05). FUBAR detects pervasive selection across branches, MEME identifies episodic selection, and SLAC estimates site‐specific substitution patterns. For functional characterization, we evaluated amino acid substitutions using PROVEAN (Choi 2012; Choi and Chan 2015; Choi et al. 2012), with scores < −2.5 indicating potentially deleterious mutations.

### 2.6 Structural Analysis of Selected Sites

Positively selected sites were mapped to protein structures using homology modeling in Swiss‐Model (Guex et al. 2009; Waterhouse et al. 2018), followed by refinement in PyMOL 2.6 (https://pymol.org/2/, accessed on 22 March 2024). Mutations were color‐coded (yellow: neutral; red: deleterious) for visualization. Transmembrane domains were predicted using Deep TMHMM v1.0.24 (Hallgren et al. 2022) to determine the structural context of selected sites within membrane‐associated proteins.

## 3 Results and Discussion

### 3.1 Mitogenome Characterization

We successfully sequenced and annotated the complete mitochondrial genomes of eight *Dopasia* specimens (Table S1; Figure S1), revealing conserved structural features typical of vertebrate mitogenomes. These assemblies ranged from 16,498 to 17,002 bp in length, consistent with other Anguidae species in GenBank. Each mitogenome contained the standard 37 genes (13 PCGs, 22 tRNAs, and 2 rRNAs) and a non‐coding control region, organized in the characteristic bidirectional transcription pattern of vertebrate mitochondria.

Gene distribution between strands followed the ancestral anguid pattern: the heavy (H) strand encoded 12 PCGs (all except ND6), both rRNAs (12S and 16S), and 14 tRNAs, while the light (L) strand contained ND6 and 8 tRNAs (tRNA‐Gln, ‐Ala, ‐Asn, ‐Cys, ‐Tyr, ‐Ser, ‐Glu, and ‐Pro). Start codons were predominantly ATG across all PCGs, with the notable exception of COXI, which initiated with GTG—a conserved feature among vertebrates.

We identified five types of termination codons: three complete (TAA, TAG, AGG) and two incomplete (TA‐ and T‐‐). These truncated stop codons, common across vertebrate mitogenomes (e.g., Tian and Guo 2022), are typically completed via post‐transcriptional polyadenylation. The overall genomic architecture, including strand asymmetry and codon usage patterns, strongly supports the conserved nature of mitochondrial organization within Anguidae while providing a robust foundation for subsequent evolutionary analyses.

Our analysis of nucleotide composition revealed remarkable conservation across the eight *Dopasia* mitogenomes (Table S2). The AT content ranged narrowly from 53.440% to 55.035%, while GC content varied slightly between 44.965% and 46.560%, consistent with the characteristic AT‐rich nature of vertebrate mitochondrial genomes (Pan et al. 2015). Strand asymmetry analysis showed positive AT‐skew values (0.115–0.148) and negative GC‐skew values (−0.369 to −0.339), indicating a pronounced bias toward adenine over thymine and cytosine over guanine in the heavy strand –a genomic signature well documented across vertebrate lineages (Pan et al. 2015).

Gene organization showed remarkable conservation of overlapping regions and intergenic spacers, including a prominent 22‐nucleotide overlap between ATP8 and ATP6 genes, a consistent 7‐nucleotide ND4–ND4L overlap, and a 5‐nucleotide ND5–ND6 overlap present in all specimens except *D. gracilis* GD04. The largest intergenic spacer (25 nucleotides) occurred between tRNA‐Asn and tRNA‐Cys, reduced to 22 nucleotides in *D. gracilis* GZ18001, while the shortest 3‐nucleotide spacers appeared between tRNA‐Ser/tRNA‐Asp and tRNA‐Glu/Cyt *b*. These structural patterns demonstrate both the conserved genomic architecture of *Dopasia* mitogenomes and minor intraspecific variations that may reflect lineage‐specific evolutionary adaptations while maintaining the fundamental organization characteristic of vertebrate mitochondrial genomes.

### 3.2 Protein‐Coding Genes

The PCGs of the eight *Dopasia* individuals exhibited highly conserved nucleotide composition (Table S2), with GC content ranging from 44.965% to 46.560% and AT content varying between 53.440% and 55.035%. Strand asymmetry analysis revealed consistent GC‐skew values (−0.369 to −0.339) and AT‐skew values (0.115–0.148), demonstrating a characteristic AT‐rich bias in the mitochondrial DNA of these specimens. These compositional features suggest strong evolutionary constraints on the mitochondrial genome, likely related to its essential role in ATP production and cellular energy metabolism (Das 2006). To investigate potential adaptive evolution, we analyzed positive selection patterns across all 13 PCGs in 36 individuals (including our eight specimens and 28 GenBank sequences, excluding outgroups).

**FIGURE 2:**
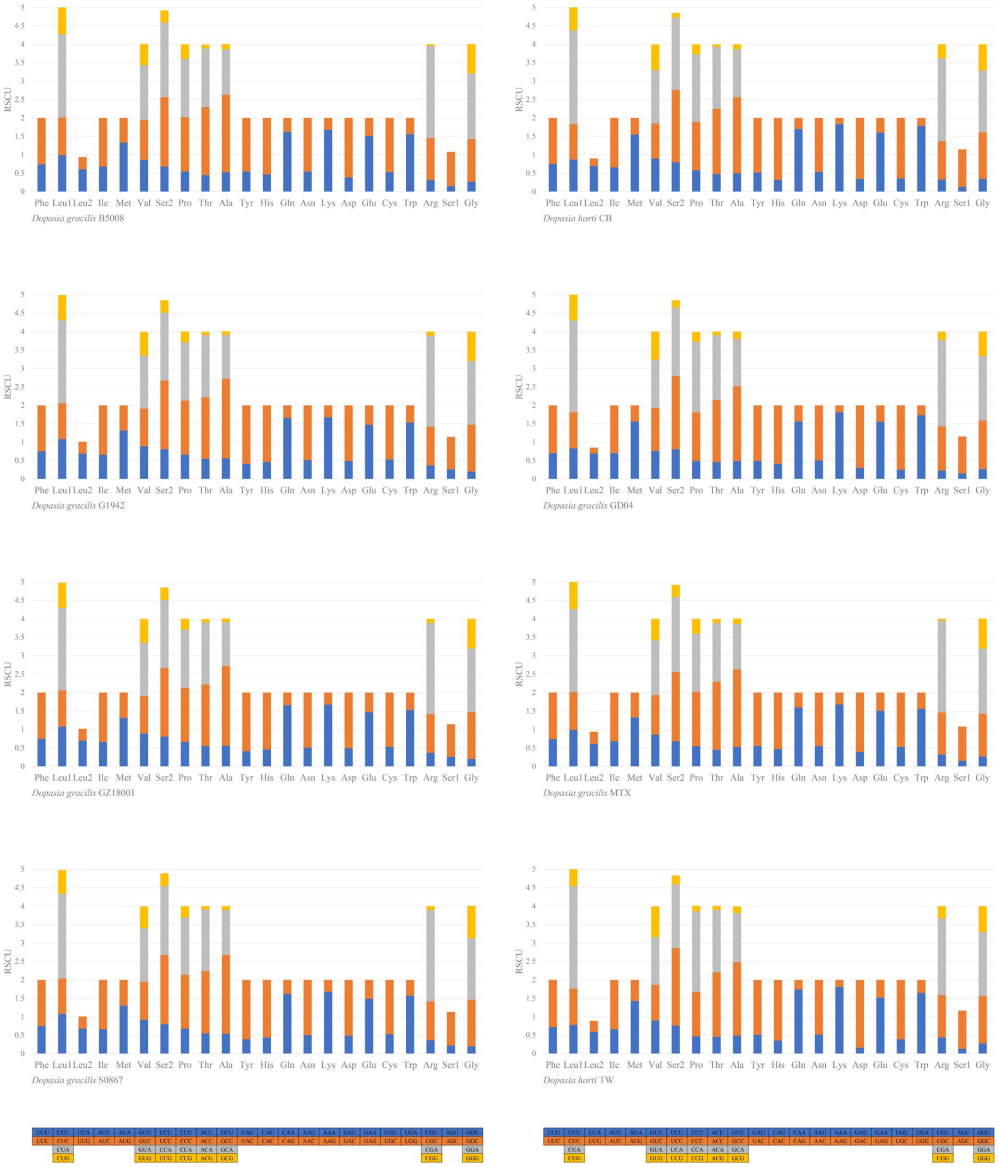
Base composition and the relative synonymous codon usage (RSCU) values for *Dopasia gracilis* (B5008, G1942, MTX, GZ18001, GD04, S0867) and *D. harti* (TW and CB). Colors indicate amino acid categories: Blue (first), orange (second), gray (third), and yellow (fourth).

Translation of mitochondrial PCGs showed consistent amino acid preferences across all specimens, with leucine, threonine, alanine, isoleucine, and serine being the most abundant (Figure S2). This pattern mirrors observations in other squamate lineages, including Lacertidae species (Tian and Guo 2022). Relative synonymous codon usage (RSCU) analysis revealed significant codon bias (Figure 2), with CGA (arginine) being the most frequent codon and ACG (threonine) the least frequent. The correlation between RSCU patterns and amino acid frequency suggests optimized translational efficiency in the mitochondrial genome, potentially reflecting adaptation to specific cellular energy demands across different populations and species.

### 3.3 Non‐Coding Region

The mitochondrial control region (CR) of all eight *Dopasia* specimens was located between tRNA‐Pro and tRNA‐Phe genes, maintaining the conserved positioning observed across vertebrate mitogenomes. CR length exhibited notable variation (1379–1830 bp), suggesting potential lineage‐specific evolutionary dynamics. Comparative analysis revealed the CR's distinct nucleotide composition, characterized by an extreme AT bias (Table S2) that significantly exceeded the AT‐richness of other mitochondrial regions. This pronounced skew primarily results from anti‐G bias at third codon positions, a well‐documented feature of vertebrate control regions that likely influences replication and transcription efficiency.

While seven specimens displayed consistent AT/GC ratios, *D. gracilis* MTX showed a slight deviation from this pattern, potentially indicating population‐specific evolutionary pressures or technical artifacts requiring verification. The CR's exceptional AT dominance and variable length may reflect its dual role in mitochondrial replication regulation and potential adaptive evolution, though further functional studies would be needed to confirm these relationships.

### 3.4 Ribosomal and Transfer RNAs

The mitochondrial rRNA genes of all eight *Dopasia* specimens maintained conserved genomic positions, with 12S rRNA located between tRNA‐Phe and tRNA‐Val (881–953 bp) and 16S rRNA positioned between tRNA‐Leu(UUR) and tRNA‐Val (1528–1557 bp). Nucleotide composition analysis revealed remarkable consistency across specimens, with thymine (19.3%–19.7%), cytosine (27.2%–27.5%), adenine (35.0%–35.6%), and guanine (17.5%–18.2%) percentages showing minimal variation. Consistent with overall mitogenome trends, both rRNA genes exhibited AT bias (54.4%–55.3% AT vs. 44.7%–45.6% GC), reflecting the characteristic nucleotide composition of vertebrate mitochondrial rRNAs (Table S2).

The 22 tRNA genes displayed length variation from 61–73 bp per tRNA, with a combined length range of 1518–1523 bp across all specimens (Table S1). Secondary structure prediction showed that 21 tRNAs conformed to the canonical cloverleaf configuration, while tRNA‐Ser (GCT) exhibited the typical loss of the dihydrouridine arm—a common feature in metazoan mitochondrial tRNAs. Comparative analysis revealed minor structural variations among specimens, including non‐Watson‐Crick base pairings and size differences in loop regions (Figure S3). These structural variations, while subtle, may reflect lineage‐specific adaptations in translational efficiency or tRNA processing.

### 3.5 Selection Pressure Analysis Across Mitochondrial PCGs

**FIGURE 3:**
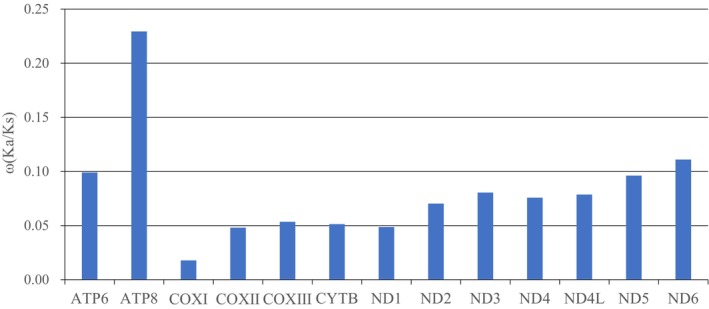
*ω* (Ka/Ks) ratios for eight mitochondrial genes in *Dopasia* species.

Our analysis of selection pressures across the 13 mitochondrial PCGs revealed strong evolutionary constraints, with all genes showing *ω* (dN/dS) ratios significantly below 1 (Figure 3). The most strongly conserved gene was COXI (*ω* = 0.0178), followed by COXII (0.0480), ND1 (0.0501), and Cyt *b* (0.0513), indicating intense purifying selection maintaining these critical respiratory chain components. In contrast, ATP8 exhibited the highest *ω* value (0.229), suggesting relatively relaxed selective constraints, consistent with patterns observed in other squamate lineages (Chen et al. 2021). The ND gene family generally showed higher *ω* values than other PCGs, potentially reflecting their more complex functional roles and greater tolerance to substitutions.

While CodeML's site models (M1a/M2a and M7/M8 comparisons) failed to detect significant positive selection (Table S3), alternative approaches revealed several candidate sites under diversifying selection. MEME identified 32 positively selected sites (*p* < 0.05), FUBAR detected 13 sites (posterior probability > 0.9), and SLAC found 6 sites (*p* < 0.05). Four codons showed consensus across all methods: positions 41 (COXIII), 3 (Cyt *b*), 26 (ND4), and 607 (ND5). Notably, codon 239 in Cyt *b* and 274 in ND2 were detected by both FUBAR and SLAC but not MEME, while codon 21 (ND4) and 605 (ND5) were identified by MEME and FUBAR (Table S4). Other codons involved in positive selection were detected by only one of the three algorithms. Codon 48 in ATP8, 260 in ND4, and codons 337 and 470 in ND5, as well as codons 103, 105, and 148 in ND6, were identified as deleterious mutations (Table S5). These discrepancies likely reflect the different statistical approaches and detection thresholds of each method.

### 3.6 Structural and Functional Implications

**FIGURE 4:**
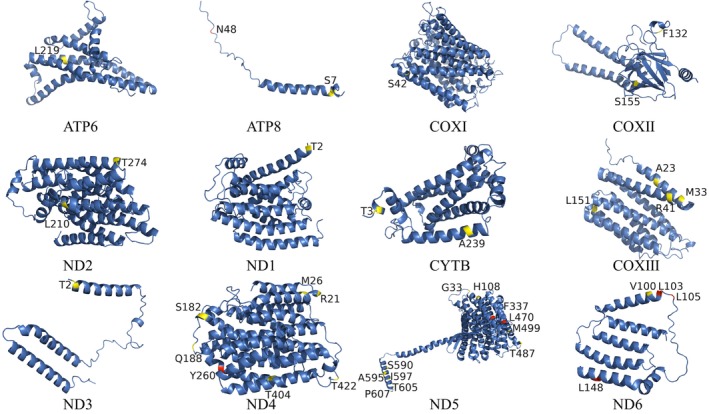
Predicted 3D structures of 13 proteins from the *Dopasia* mitogenome, with positive selection sites (Datamonkey) highlighted. Deleterious mutations are shown in red and neutral mutations in yellow (PROVEAN).

**FIGURE 5:**
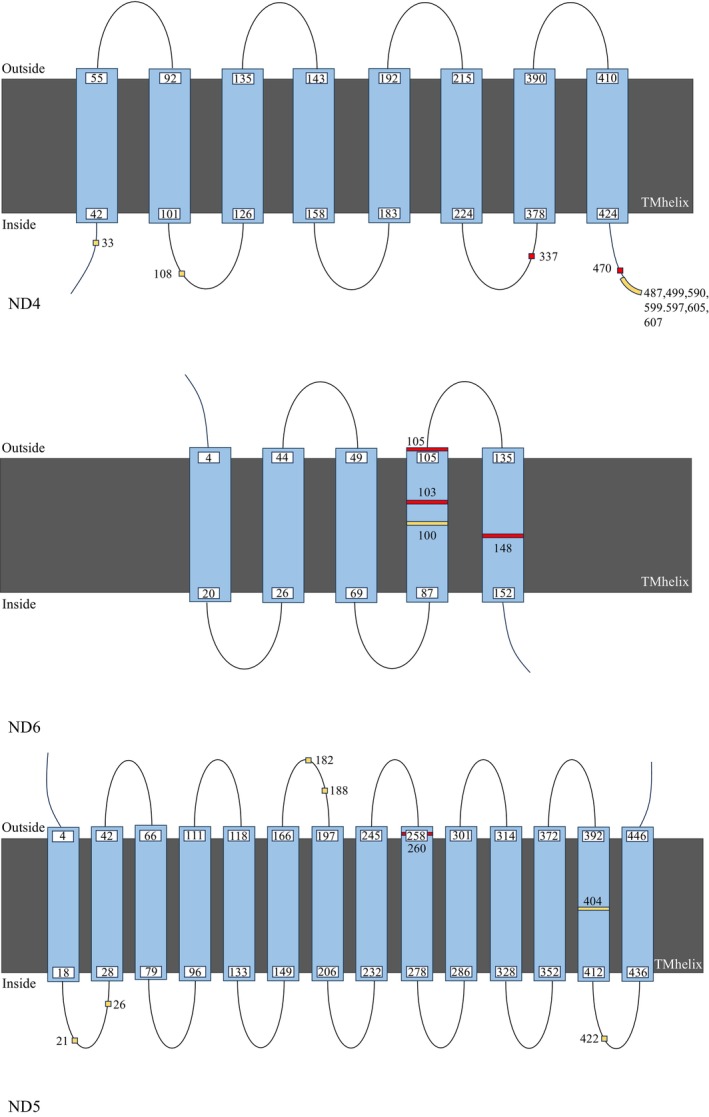
Predicted transmembrane helix topologies for ND4, ND5, and ND6 (Deep TMHMM). Positive selection sites (Datamonkey) are highlighted: Red for deleterious mutations and yellow for neutral mutations (PROVEAN).

Mapping the positively selected sites to protein structures revealed several important patterns (Figures 4 and 5). Selected sites were distributed across all structural domains (α‐helices 46%, β‐sheets 27%, coils 27%), with notable clustering in functional regions. COXIII and ND6 showed centralized distributions in catalytic cores, while ND4 and ND5 sites were more dispersed. Membrane topology analysis demonstrated that ND5 sites were exclusively membrane‐embedded, ND6 sites localized to transmembrane helices, and ND4 sites spanned all compartments. Several deleterious mutations identified by PROVEAN were located in functionally critical domains, including the Mrp antiporter region of ND5 (Table S5), suggesting potential impacts on proton translocation efficiency (Morino et al. 2014).

### 3.7 Geographic Patterns of Molecular Evolution

Analysis of 38 positively selected sites revealed distinct geographic patterns of molecular evolution within the genus *Dopasia* (Table S6), with characteristic amino acid variants differentiating these species from related taxa. All examined *Dopasia* specimens from China shared several conserved residues, including I219 in ATP6 (absent only in *Pseudopus apodus* ), L151 in COXIII, and T2 in ND1 and ND3 (divergent in *Abronia graminea* at ND3). We also identified polymorphic sites showing intragenus variation, particularly in Complex I genes: ND4 exhibited R/G21, S/M26, and S/F182 variants; ND5 showed variation at positions 33 (G), 108 (H), 470 (F/L/T), 590 (S), and 607 (P/S/T); while ND6 displayed L/W103 and L/C105 polymorphisms. These variants are concentrated in Chinese *Dopasia* populations (except *D. sokolovi* ) and cluster in key respiratory complexes, suggesting potential adaptation to local environmental pressures on mitochondrial function—either through relaxed constraints or balancing selection across habitats.

Our analysis also revealed distinct patterns of mitochondrial variation across taxa and geography. *Anguis colchica* displayed unique amino acid substitutions including leucine at position 33 of COXIII, while showing valine/isoleucine polymorphism at position 499 of ND5 (except in *Abronia graminea* ) and methionine at position 487 of ND5. *Pseudopus apodus* was characterized by threonine at position 210 of ND2 and methionine at position 499 of ND5, while *D. harti* (KF279681) varied at these positions and uniquely possessed serine at position 595 of ND5 (absent in *D. sokolovi* ).

Within the genus *Dopasia*, we observed population‐specific molecular signatures. The arginine variant at position 41 of COXIII specifically characterizes *D. gracilis* populations from Guangxi and Yunnan (vouchers G1942, S0867; accessions MN661343, KJ941042). Additional diagnostic variants for *D. gracilis* include threonine at position 274 of ND2 and methionine at position 499 of ND5, found in specimens from Guizhou, Guangxi, and Yunnan. Tibetan (B5008, MTX) and Guangxi populations show nearly identical profiles, differing only at four specific positions (COXI‐42, ND1‐2, ND4‐26, ND5‐595), while Yunnan specimens form a distinct cluster with consistent variants.

Geographic patterns of mitochondrial variation were also evident in European *Anguis fragilis* . While most populations share conserved sequences, Spanish specimens (OP493519, EU443256) show distinct variants at positions 404 of ND4, 155 of COXII, and 41 of COXIII compared to other *A. fragilis* populations (OP493518, MN122840). Interestingly, position 337 of ND5 does not follow this geographic pattern, suggesting different evolutionary pressures at this site.

These findings align with documented cases of mitochondrial adaptation across vertebrates, including fish (Zhao et al. 2022), bats (Zhang et al. 2021), and turtles (Sahoo et al. 2023). The concentration of population‐specific variants in Chinese *Dopasia* suggests that geographic isolation may have driven the fixation of mitochondrial mutations, potentially facilitating local environmental adaptation through modifications to oxidative phosphorylation efficiency. The persistence of these molecular signatures in phylogenetic analyses underscores their evolutionary significance and provides insights into the role of mitogenome evolution in species diversification.

### 3.8 Phylogenetic Relationships and Evolutionary Implications

**FIGURE 6:**
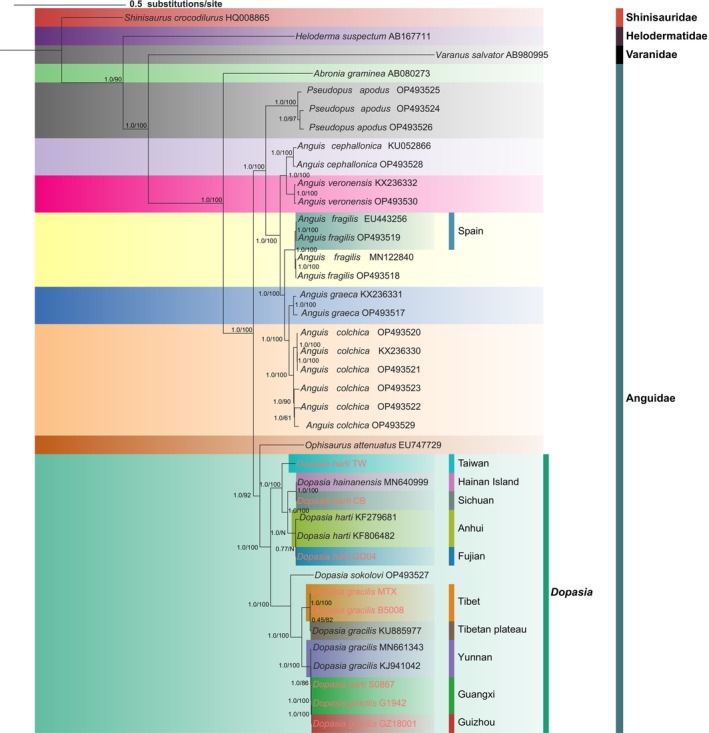
50% majority‐rule consensus tree of Anguidae from partitioned Bayesian analysis of concatenated PCGs (36 ingroup + 3 outgroup taxa). Node values are Bayesian posterior probabilities (PP) and ML ultrafast bootstrap (UFBoot) support; N marks nodes absent in the ML tree. Branch lengths represent posterior means. Species names include GenBank accession numbers; families and geographic locations are indicated. Eight *Dopasia* individuals from this study are highlighted in red.

Phylogenetic analyses using both Bayesian Inference (BI) and Maximum Likelihood (ML) methods produced highly congruent tree topologies, with strong nodal support (UFBoot > 95% for ML) for most relationships (Figure 6). The genus *Anguis* was recovered as monophyletic and formed a clade with *Pseudopus apodus* , which together served as sister to a clade containing *Dopasia* and *Ophisaurus attenuatus* . These groups collectively showed a sister relationship to *Abronia graminea* , consistent with established relationships in the Anguidae family.

The genus *Dopasia* exhibited clear phylogenetic structure, with *D. gracilis* and *D. sokolovi* forming a clade sister to *D. harti* . *D. hainanensis* is nested within the *D. harti* clade, supporting its recent synonymization under *D. harti* (Li et al. 2024). Although *D. harti* contains several mitochondrial lineages, divergence is shallow and lacks corresponding morphological or geographic structure. Li et al. (2024) reported no significant morphological differences between topotypic specimens in diagnostic characters (dorsal scales between lateral grooves, ear size, and dorsal transverse banding), and Cyt *b* gene divergence is only 4.96%. Given the generally low morphological differentiation in *Dopasia*, these results support synonymizing *D. hainanensis* with *D. harti* .

The phylogenetic analyses revealed clear geographic structuring across species, with *D. harti* populations forming distinct lineages (CB, GD04/KF806482/KF279681, and TW clades), *D. gracilis* showing regional differentiation (Tibetan MTX/B5008, Yunnan MN661343/KJ941042, and Guangxi G1942/S0867 clusters), and *Anguis fragilis* exhibiting continental‐scale divergence between Spanish (OP493519/EU443256) and other European populations (OP493518/MN122840). These patterns demonstrate how geographic isolation has shaped genetic divergence through mechanisms like mountain barriers, river systems, and Pleistocene refugia, with mitochondrial lineages closely tracking spatial distributions and highlighting the role of allopatric processes in Anguidae diversification.

These phylogenetic patterns align with the geographic correlations observed in our positive selection analysis, suggesting that both neutral evolutionary processes and adaptive evolution have contributed to the genetic differentiation of these species. The strong geographic structuring within species complexes supports models of ecological speciation, where habitat differences and geographic isolation drive genetic divergence. Our results are largely consistent with previous molecular studies of Anguidae (Cai, Guo, and Jiang 2020; Cai, Guo, Song, and Chen 2020; Lavin and Girman 2019; Zhan et al. 2024), reinforcing the robustness of these evolutionary relationships while providing new insights into intraspecific variation within *Dopasia*. The congruence between phylogenetic relationships and geographic distributions highlights the importance of considering both evolutionary history and contemporary population structure in understanding speciation processes in this group.

## 4 Conclusions

This study provides the first complete mitochondrial genomes for eight *Dopasia* individuals, revealing conserved vertebrate mitogenome architecture with notable lineage‐specific variations. Phylogenetic reconstruction using concatenated PCGs (13 genes, 36 specimens) resolved relationships within Anguidae, demonstrating that while purifying selection dominates mitochondrial evolution, discrete sites show signatures of positive selection. Crucially, these selected sites exhibit geographic patterning that mirrors phylogenetic divergences, suggesting local adaptation to environmental pressures. The concentration of adaptive mutations in key respiratory genes (particularly ND4, ND5, and COXIII) implies functional optimization of oxidative phosphorylation under different ecological conditions. These findings advance our understanding of how mitogenome evolution contributes to ecological diversification in squamate reptiles.

## Author Contributions

**Bo Cai:** data curation (equal), funding acquisition (equal), investigation (lead), project administration (lead), resources (lead), writing – review and editing (equal). **Chengguanyu Liu:** data curation (equal), formal analysis (lead), methodology (equal), writing – original draft (lead). **Gaus Shang:** investigation (equal), resources (equal). **Yingyong Wang:** investigation (equal), resources (equal). **Sikan Chen:** investigation (equal), resources (equal). **Dong Liang:** investigation (equal), resources (equal). **Wei Zeng:** investigation (equal), resources (equal). **Shichao Zheng:** investigation (equal), resources (equal). **Xianguang Guo:** formal analysis (equal), funding acquisition (lead), methodology (equal), project administration (equal), supervision (equal), writing – original draft (equal).

## Funding

This work was supported by the Science & Technology Department of Sichuan Province (Grant No. 2025ZNSFSC0249), the Special Investigation on Amphibians and Reptiles in the Chengdu Section of Giant Panda National Park (2025014) and the Society of Entrepreneurs and Ecology (China SEE) Youth Scholar Research Fund Project (Project No. SEE‐B‐3709).

## Conflicts of Interest

The authors declare no conflicts of interest.

## Supporting information


**Figure S1:** Mitochondrial genome map of *Dopasia gracilis* B5008. Genes on the light and heavy strands are shown inside and outside the circle, respectively, with arrows indicating transcription direction. tRNA genes are colored blue and labeled with three‐letter amino acid codes. Maps for other individuals analyzed are similar.

**Figure S2:** Amino acid frequencies in eight *Dopasia* mitogenome.

**Figure S3:** Secondary structures of 22 mitochondrial tRNAs for eight *Dopasia* individuals, predicted by tRNAScan‐SE 2.0.

**Figure S4:** Deep TMHMM‐predicted transmembrane helix topologies for ND1, ND2, ND3, COXI, COXII, COXIII, Cyt b, ATP6, and ATP8. Positive selection sites (Datamonkey) are highlighted: red for deleterious mutations and yellow for neutral mutations (PROVEAN).

**Table S1:** Codon and anticodon usage for eight complete mitogenomes of *Dopasia gracilis* (B5008, G1942, MTX, GZ18001, GD04, S0867) and *D. harti* (TW, CB).

**Table S2:** Base composition of eight *Dopasia* mitogenomes.

**Table S3:** CodeML site model specifications.

**Table S4:** Candidate codon sites under positive selection from three Datamonkey tests.

**Table S5:** Neutral and deleterious mutation sites predicted in PROVEAN.

**Table S6:** Amino acid composition of positive selection sites (Datamonkey).

## Data Availability

All materials are included in the main text and the Supporting Information.
