# Correction to “Complete Mitochondrial Genome Sequencing of Asian Glass Lizards (Anguidae: *Dopasia*): Comparative Analysis With Limbless Anguids and New Insights Into the Adaptive Evolution of Protein‐Coding Genes”

**DOI:** 10.1002/ece3.73122

**Published:** 2026-02-15

Cai, B., C. Liu, G. Shang, et al. 2025. “Complete Mitochondrial Genome Sequencing of Asian Glass Lizards (Anguidae: *Dopasia*): Comparative Analysis With Limbless Anguids and New Insights Into the Adaptive Evolution of Protein‐Coding Genes.” *Ecology and Evolution* 15, no. 12: e72811. https://doi.org/10.1002/ece3.72811

In the published article, the GenBank accession number of Table 1 was incomplete. The accession numbers for the eight newly sequenced *Dopasia* are as follows:

B5008: PQ963862; G1942: PV083567; MTX: PV083570: GZ18001: PV083569; GD04: PV083568: S0867: PV083571; TW: PV083572; CB: PV083566.

The updated Table 1 is shown below.

**TABLE 1:** Taxon information and GenBank accession numbers for Anguidae and two outgroup species.

Taxon	Family	Accession number	Length (bp)	Country	Locality	References
*Dopasia*	Anguidae					
*Dopasia gracilis* B5008	Anguidae	PQ963862	17,218	China	Tibet	This study
*Dopasia gracilis* G1942	Anguidae	PV083567	17,241	China	Guangxi	This study
*Dopasia gracilis* MTX	Anguidae	PV083570	16,823	China	Tibet	This study
*Dopasia gracilis* GZ18001	Anguidae	PV083569	16,775	China	Guizhou	This study
*Dopasia gracilis* GD04	Anguidae	PV083568	17,070	China	Fujian	This study
*Dopasia gracilis* S0867	Anguidae	PV083571	17,272	China	Guangxi	This study
*Dopasia harti* TW	Anguidae	PV083572	17,069	China	Taiwan	This study
*Dopasia harti* CB	Anguidae	PV083566	17,000	China	Sichuan	This study
*Dopasia hainanensis*	Anguidae	MN640999	17,000	China	Hainan	Gvoždík et al. (2023)
*Dopasia harti*	Anguidae	KF806482	17,571	China	Anhui	Yan (2015)
*Dopasia harti*	Anguidae	KF279681	17,163	China	Anhui	Pan et al. (2015)
*Dopasia gracilis*	Anguidae	MN661343	17,133	China	Yunnan	Cai, Guo, and Jiang (2020), Cai, Guo, Song, and Chen (2020)
*Dopasia gracilis*	Anguidae	KJ941042	17,788	China	Yunnan	Yan (2015)
*Dopasia gracilis*	Anguidae	KU885977	16,777	China	Tibetan Plateau	Fang (Unpubl. data)
*Dopasia sokolovi*	Anguidae	OP493527	17,137	Vietnam	Bidoup‐Nui Ba	Gvoždík et al. (2023)
*Anguis*	Anguidae					
*Anguis graeca*	Anguidae	OP493517	17,103	Greece	Mornos River	Gvoždík et al. (2023)
*Anguis graeca*	Anguidae	KX236331	19,297	N.A.	N.A.	Strzala (Unpubl. data)
*Anguis fragilis*	Anguidae	OP493518	17,096	Czech	Rep. Zelízy	Gvoždík et al. (2023)
*Anguis fragilis*	Anguidae	OP493519	17,103	Spain	Torla‐Ordesa	Gvoždík et al. (2023)
*Anguis fragilis*	Anguidae	EU443256	17,479	Spain	Vilarmiel Lugo	Albert et al. (2009)
*Anguis fragilis*	Anguidae	MN122840	17,134	N.A.	N.A.	Margaryan (Unpubl. data)
*Anguis colchica*	Anguidae	OP493520	17,383	Czech	Rep. Ostravice	Gvoždík et al. (2023)
*Anguis colchica*	Anguidae	OP493521	17,390	Lithuania	Marcinkony	Gvoždík et al. (2023)
*Anguis colchica*	Anguidae	OP493522	17,420	Iran	Motalla Sara‐ye Lemir	Gvoždík et al. (2023)
*Anguis colchica*	Anguidae	OP493523	17,125	Bulgaria	Sinemore	Gvoždík et al. (2023)
*Anguis colchica*	Anguidae	OP493529	17,168	Georgia	Telavi	Gvoždík et al. (2023)
*Anguis colchica*	Anguidae	KX236330	17,097	Poland	Bieszczady Mts. Zatwarnica village	Strzała, Grochowalska, Najbar, Najbar, and Jablonski (2017)
*Anguis veronensis*	Anguidae	OP493530	17,186	Italy	Roccagnano	Gvoždík et al. (2023)
*Anguis veronensis*	Anguidae	KX236332	17,322	Italy	Bergamo	Strzała, Grochowalska, Najbar, Crottini, et al. (2017)
*Anguis cephallonica*	Anguidae	OP493528	17,224	Greece	Gialova	Gvoždík et al. (2023)
*Anguis cephallonica*	Anguidae	KU052866	17,208	Greece	Peloponnese Peninsula	Strzała et al. (2016)
*Pseudopus*	Anguidae					
*Pseudopus apodus*	Anguidae	OP493524	16,701	Georgia	Dedop'lis Tskaro	Gvoždík et al. (2023)
*Pseudopus apodus*	Anguidae	OP493526	16,901	Albania	Zogaj	Gvoždík et al. (2023)
*Pseudopus apodus*	Anguidae	OP493525	17,104	Israel	Hare Gilboa'	Gvoždík et al. (2023)
*Ophisaurus*	Anguidae					
*Ophisaurus attenuatus*	Anguidae	EU747729	14,628	N.A.	N.A.	Castoe et al. (2008)
*Abronia*	Anguidae					
*Abronia graminea*	Anguidae	AB080273	16,016	N.A.	N.A.	Kumazawa and Nishida (1995)
**Outgroups**						
*Heloderma suspectum*	Helodermatidae	AB167711	16,846	Japan	Nagoya Higashiyama Zoo	Kumazawa (2007)
*Shinisaurus crocodilurus*	Shinisauridae	HQ008865	16,585	N.A.	N.A.	Li et al. (2012)
*Varanus salvator*	Varanidae	AB980995	17,536	Thailand	Nakhon Ratchasima Zoo	Chaiprasertsri et al. (2013)

We apologize for this error.

