# Supplementary figures and images for: Complete Mitochondrial Genome Sequencing of Asian Glass Lizards (Anguidae: Dopasia): Comparative Analysis With Limbless Anguids and New Insights Into the Adaptive Evolution of Protein‐Coding Genes
Source: Ecol Evol. 2025 Dec 25;15(12):e72811. doi: 10.1002/ece3.72811 (PMC12740153; doi:10.1002/ece3.72811)

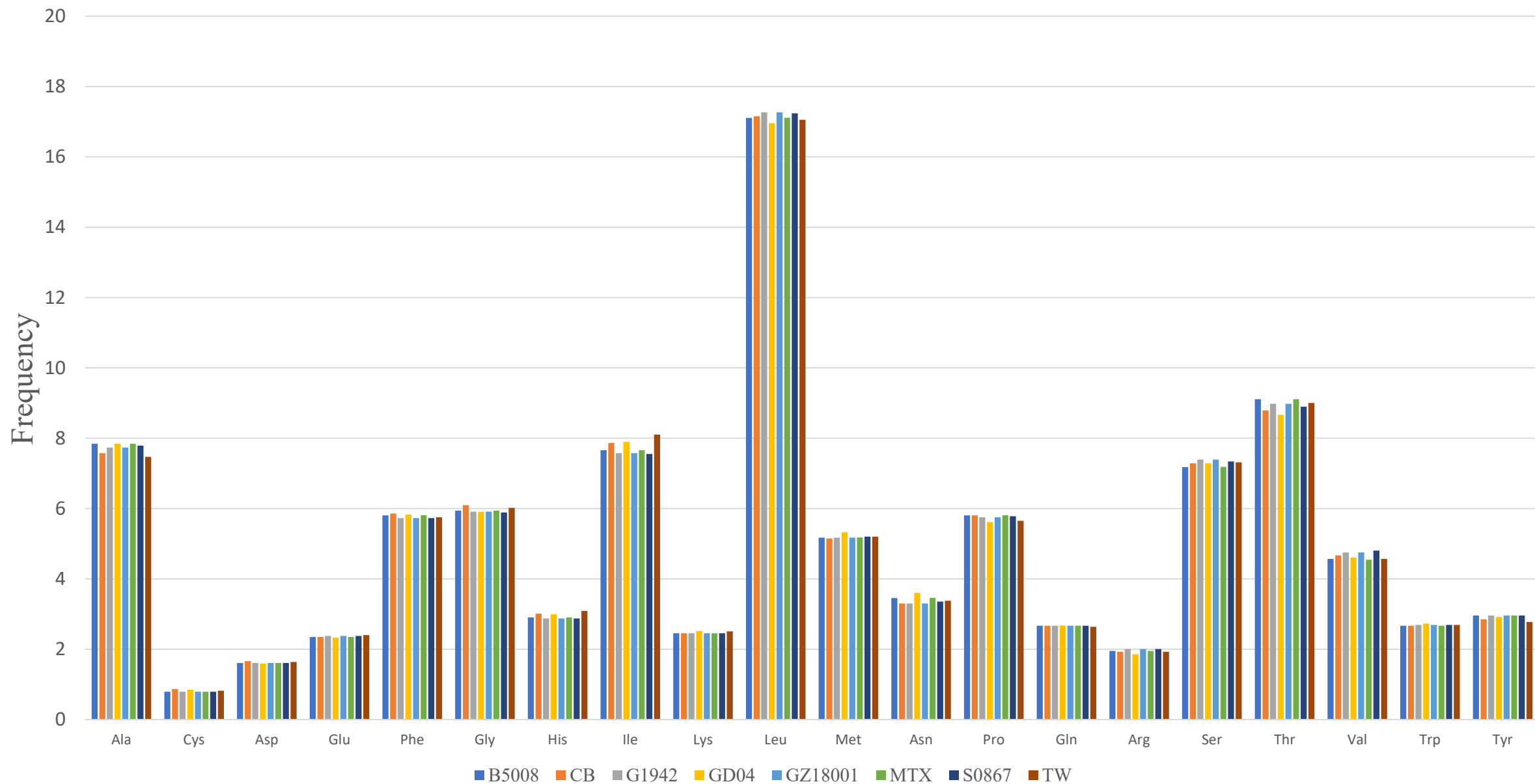

Supplement: Supplementary file 2 — Figure S2: Amino acid frequencies in eight Dopasia mitogenome. [file ECE3-15-e72811-s003.pdf]

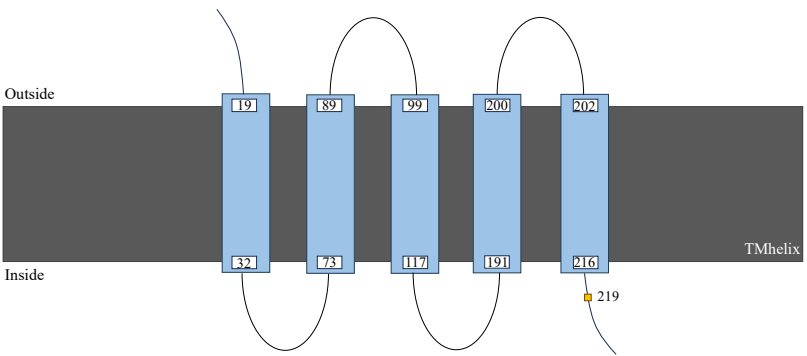

ATP6

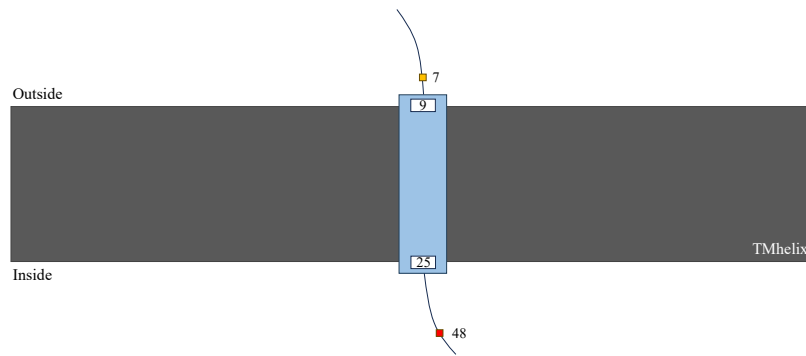

ATP8

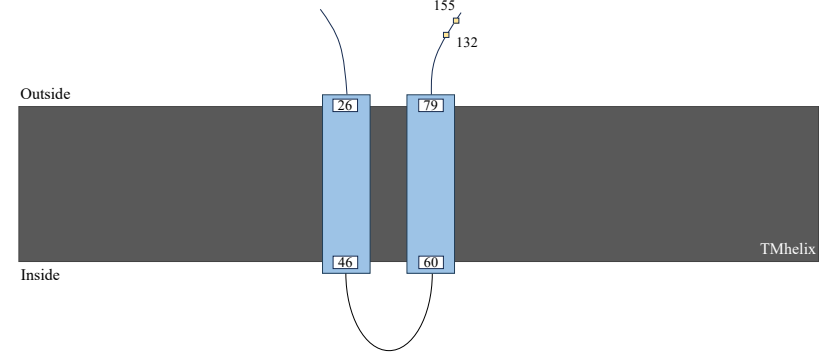

COXII

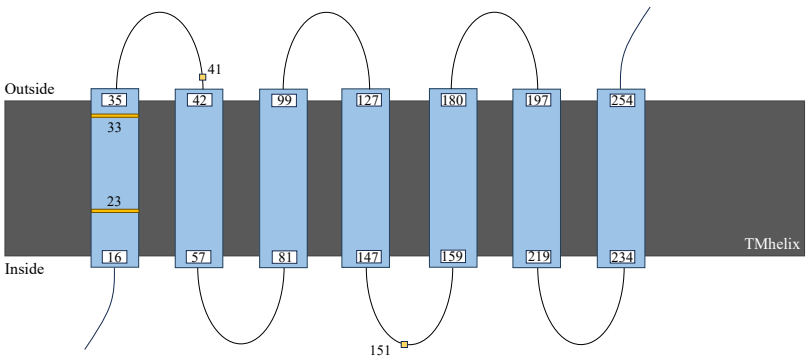

COXIII

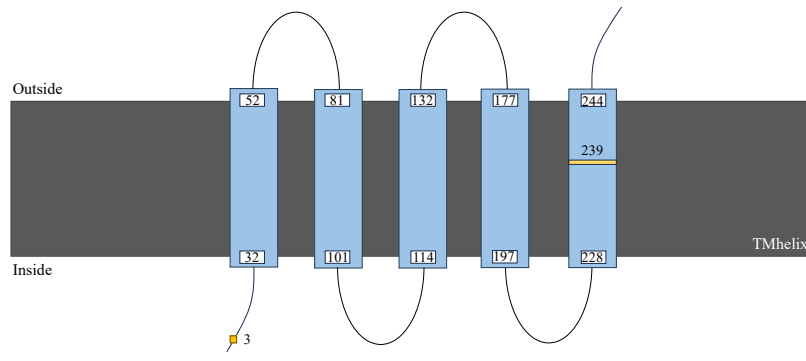

CYTB

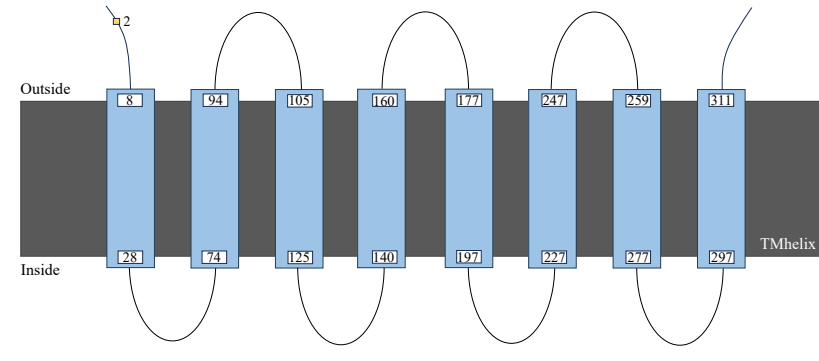

ND1

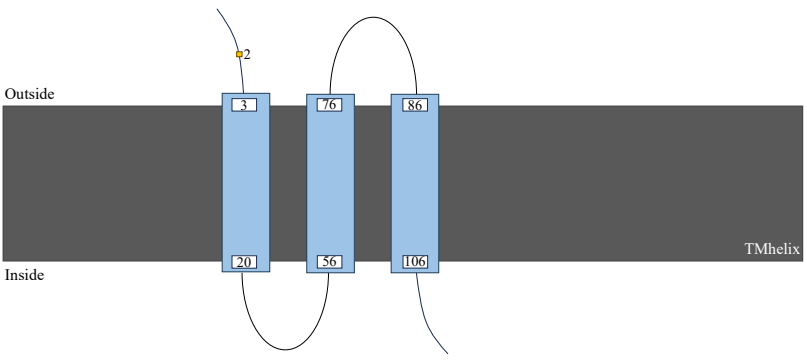

ND3

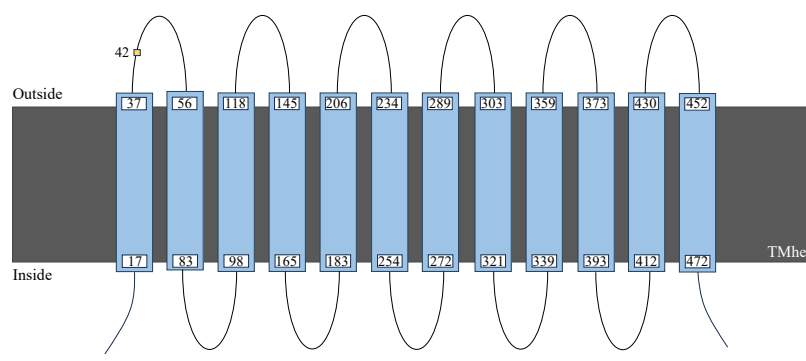

COXI

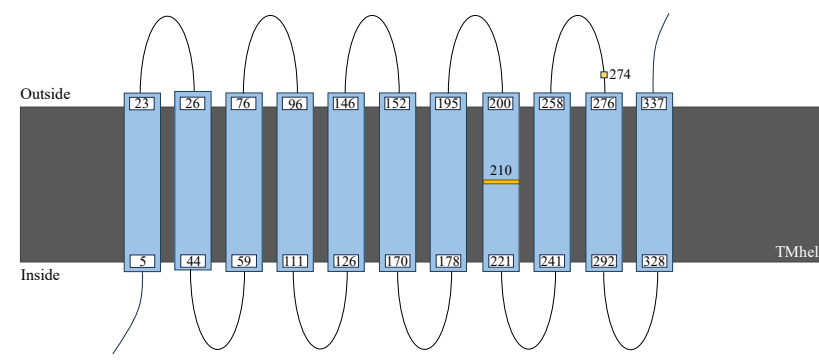

ND2

Supplement: Supplementary file 4 — Figure S4: Deep TMHMM‐predicted transmembrane helix topologies for ND1, ND2, ND3, COXI, COXII, COXIII, Cyt b, ATP6, and ATP8. Positive selection sites (Datamonkey) are highlighted: red for deleterious mutations and yellow for neutral mutations (PROVEAN). [file ECE3-15-e72811-s008.pdf]
