# Supplementary material for: Complete Mitochondrial Genome Sequencing of Asian Glass Lizards (Anguidae: Dopasia): Comparative Analysis With Limbless Anguids and New Insights Into the Adaptive Evolution of Protein‐Coding Genes
Source: Ecol Evol. 2025 Dec 25;15(12):e72811. doi: 10.1002/ece3.72811 (PMC12740153; doi:10.1002/ece3.72811)
Supplement: Supplementary file 3 — Figure S3: Secondary structures of 22 mitochondrial tRNAs for eight Dopasia individuals, predicted by tRNAScan‐SE 2.0. [file ECE3-15-e72811-s010.pdf]

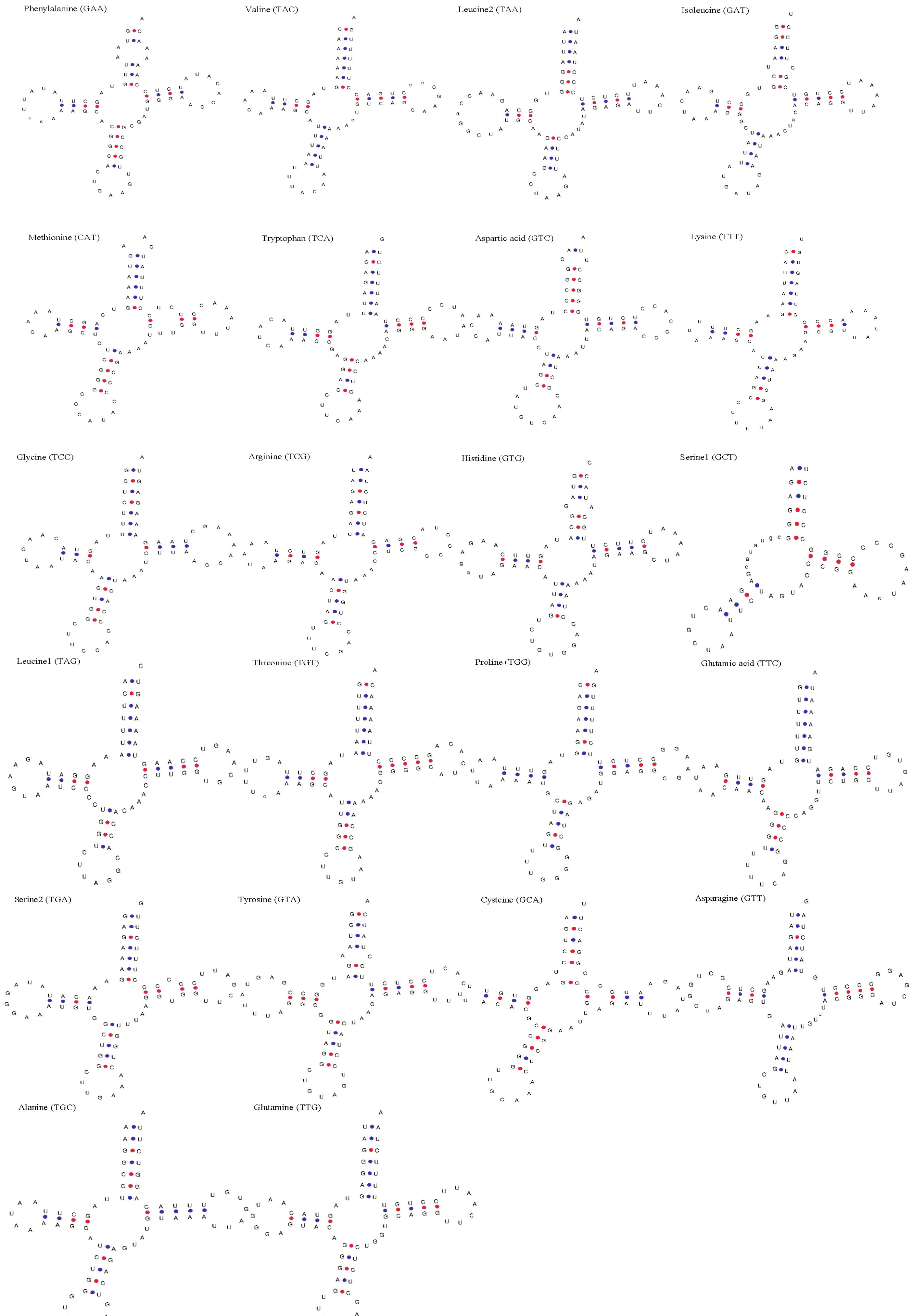
Phenylalanine (GAA)
Valine (TAC)
Leucine2 (TAA)
Isoleucine (GAT)
Methionine (CAT)
Tryptophan (TCA)
Aspartic acid (GTC)
Lysine (TTT)
Glycine (TCC)
Arginine (TCG)
Histidine (GTG)
Serine1 (GCT)
Leucine1 (TAG)
Threonine (TGT)
Proline (TGG)
Glutamic acid (TTC)
Serine2 (TGA)
Tyrosine (GTA)
Cysteine (GCA)
Asparagine (GTT)
Alanine (TGC)
Glutamine (TTG)

B5008

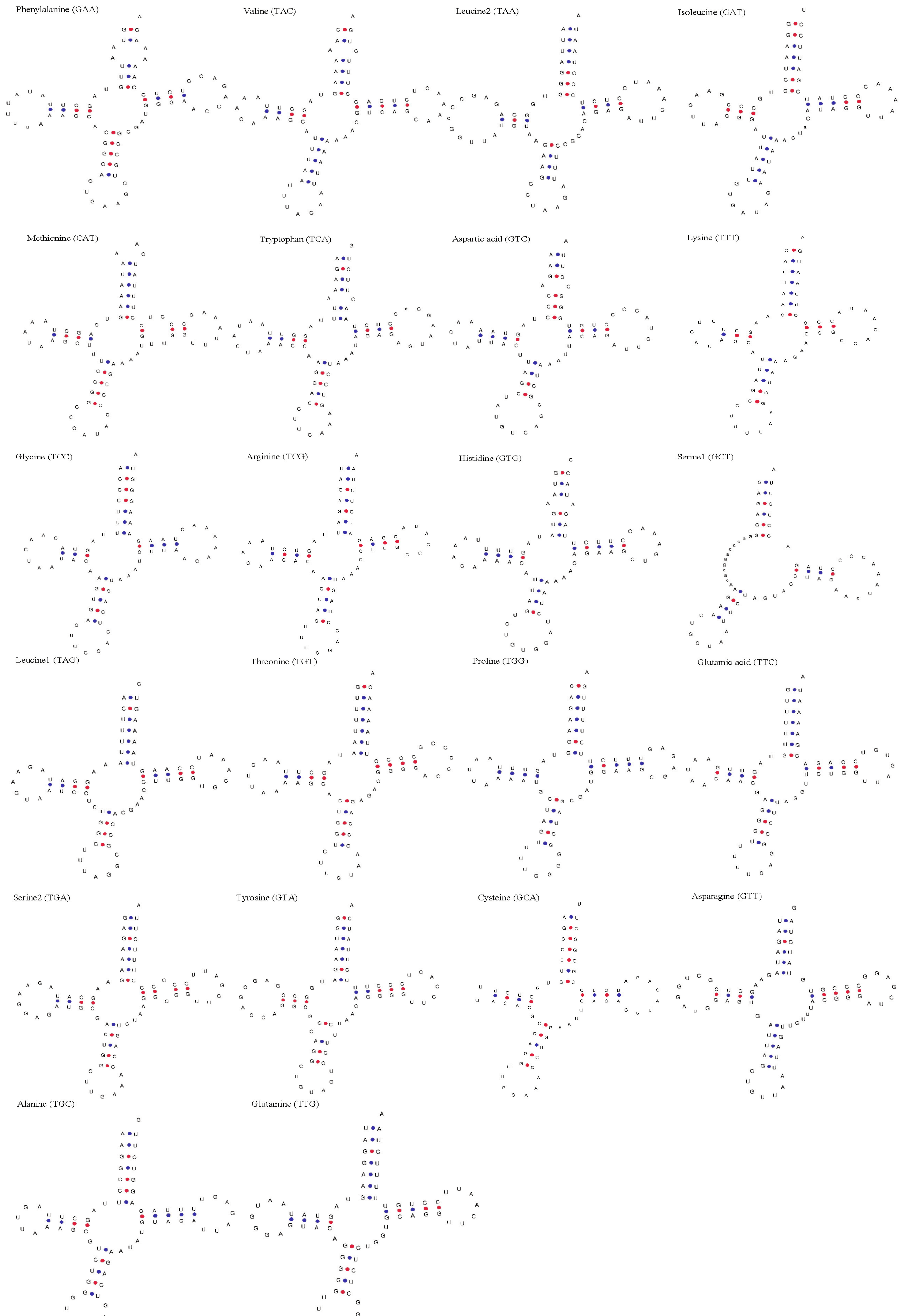
Phenylalanine (GAA)
Valine (TAC)
Leucine2 (TAA)
Isoleucine (GAT)
Methionine (CAT)
Tryptophan (TCA)
Aspartic acid (GTC)
Lysine (TTT)
Glycine (TCC)
Arginine (TCG)
Histidine (GTG)
Serine1 (GCT)
Leucine1 (TAG)
Threonine (TGT)
Proline (TGG)
Glutamic acid (TTC)
Serine2 (TGA)
Tyrosine (GTA)
Cysteine (GCA)
Asparagine (GTT)
Alanine (TGC)
Glutamine (TTG)

CB

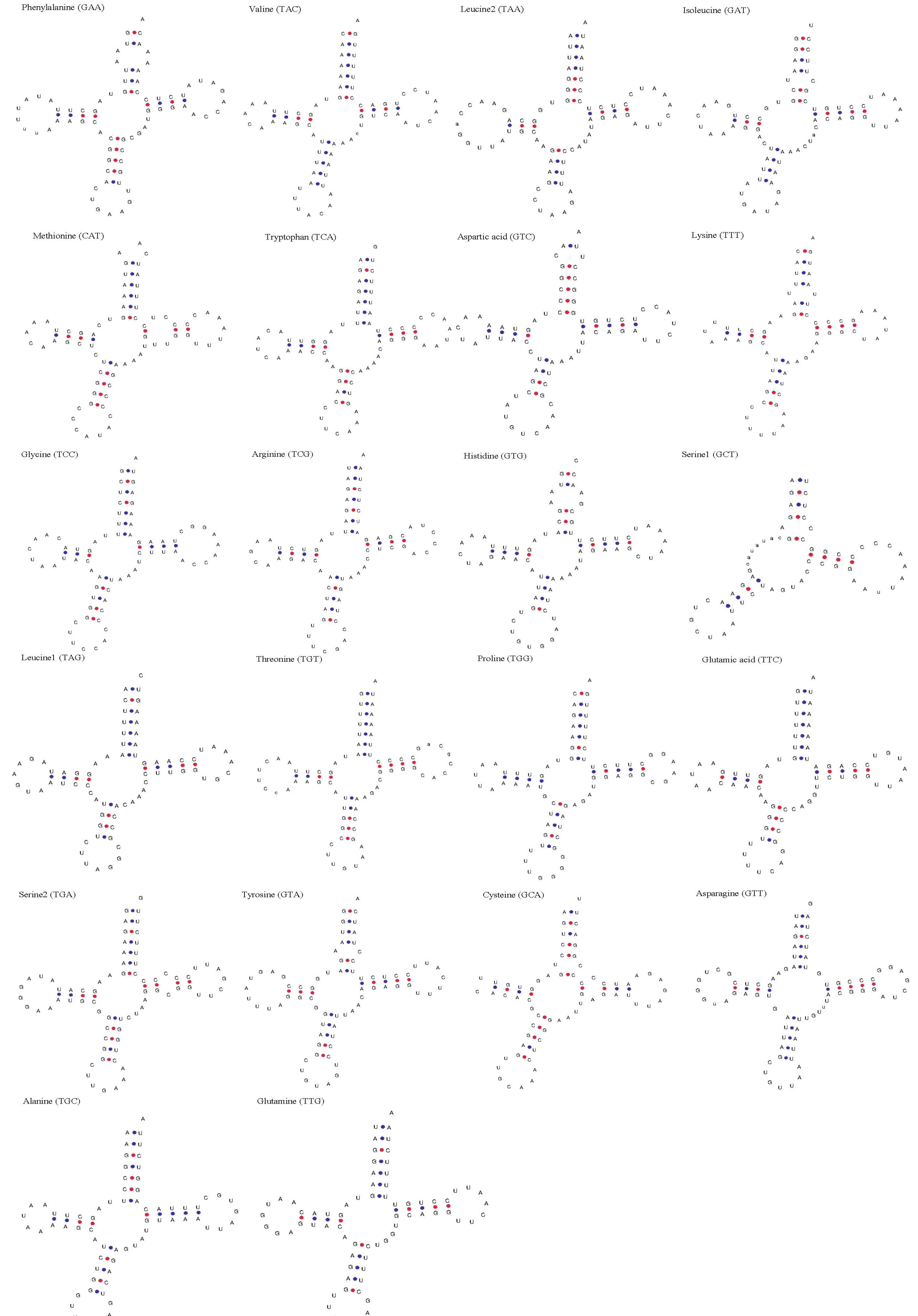
Phenylalanine (GAA)
Valine (TAC)
Leucine2 (TAA)
Isoleucine (GAT)
Methionine (CAT)
Tryptophan (TCA)
Aspartic acid (GTC)
Lysine (TTT)
Glycine (TCC)
Arginine (TCG)
Histidine (GTG)
Serine1 (GCT)
Leucine1 (TAG)
Threonine (TGT)
Proline (TGG)
Glutamic acid (TTC)
Serine2 (TGA)
Tyrosine (GTA)
Cysteine (GCA)
Asparagine (GTT)
Alanine (TGC)
Glutamine (TTG)

G1942

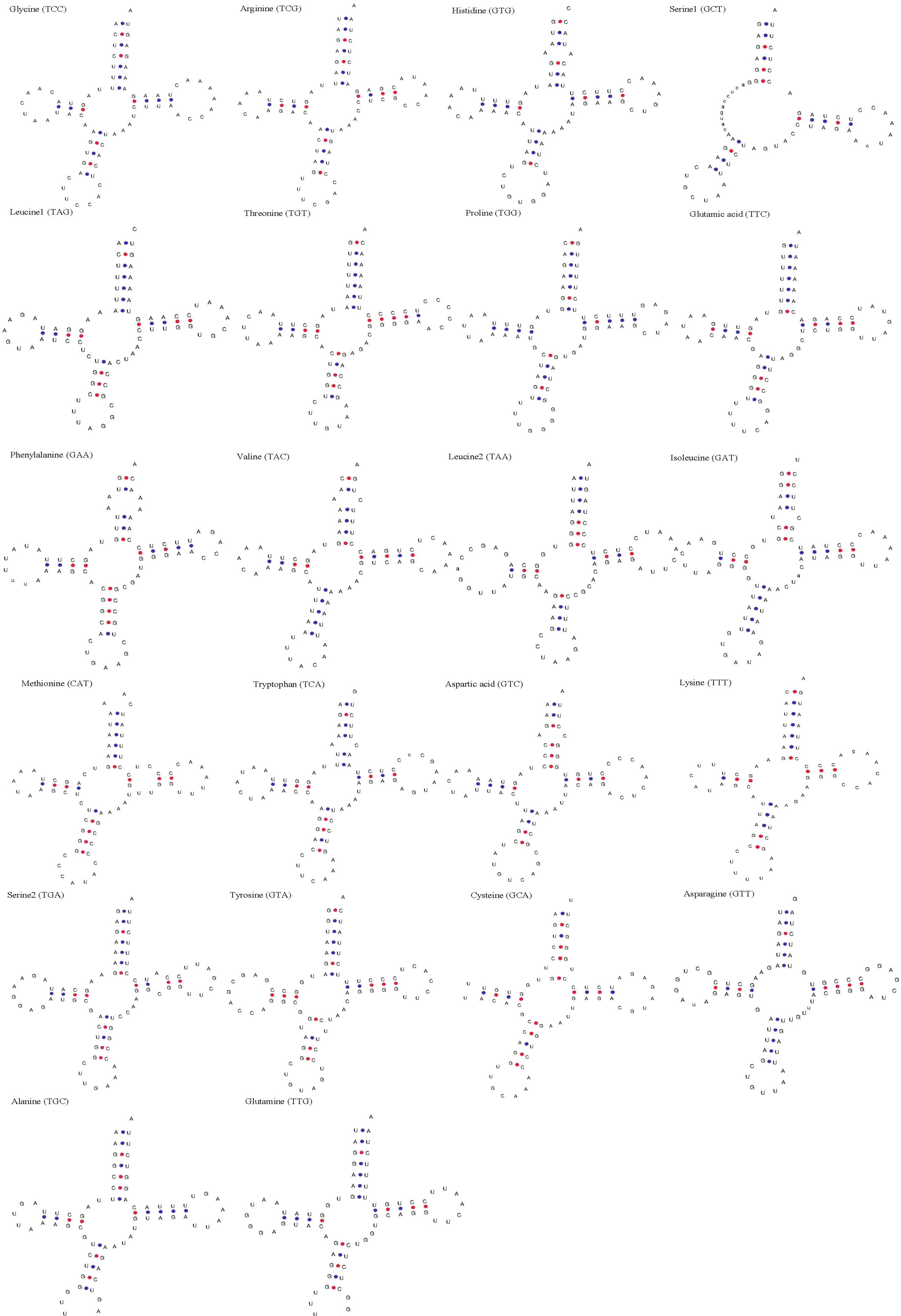
Glycine (TCC)
Arginine (TCG)
Histidine (GTG)
Serine1 (GCT)
Leucine1 (TAG)
Threonine (TGT)
Proline (TGG)
Glutamic acid (TTC)
Phenylalanine (GAA)
Valine (TAC)
Leucine2 (TAA)
Isoleucine (GAT)
Methionine (CAT)
Tryptophan (TCA)
Aspartic acid (GTC)
Lysine (TTT)
Serine2 (TGA)
Tyrosine (GTA)
Cysteine (GCA)
Asparagine (GTT)
Alanine (TGC)
Glutamine (TTG)

GD04

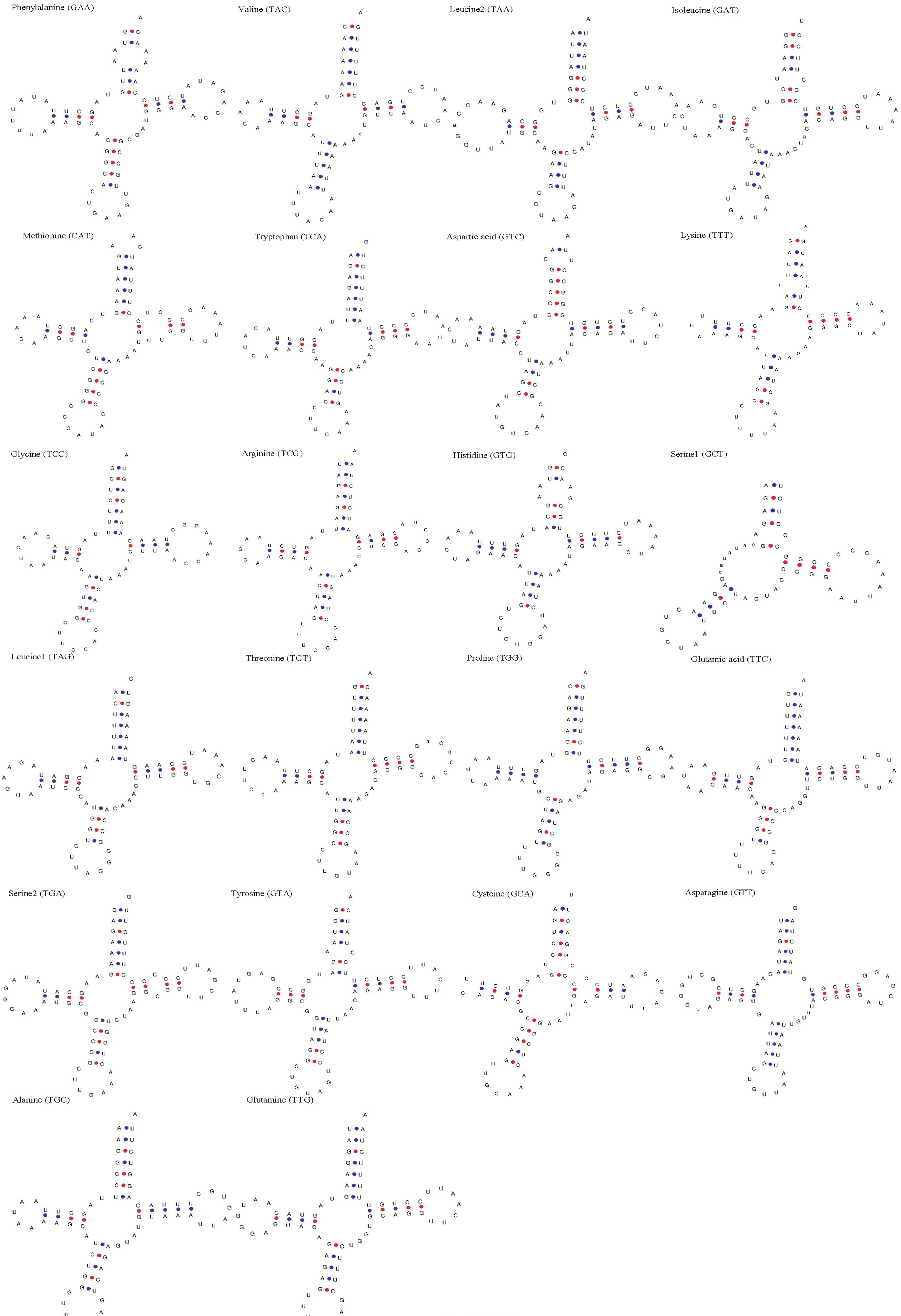
Phenylalanine (GAA)
Valine (TAC)
Leucine2 (TAA)
Isoleucine (GAT)
Methionine (CAT)
Tryptophan (TCA)
Aspartic acid (GTC)
Lysine (TTT)
Glycine (TCC)
Arginine (TCG)
Histidine (GTG)
Serine1 (GCT)
Leucine1 (TAG)
Threonine (TGT)
Proline (TGG)
Glutamic acid (TTC)
Serine2 (TGA)
Tyrosine (GTA)
Cysteine (GCA)
Asparagine (GTT)
Alanine (TGC)
Glutamine (TTG)

GZ18001

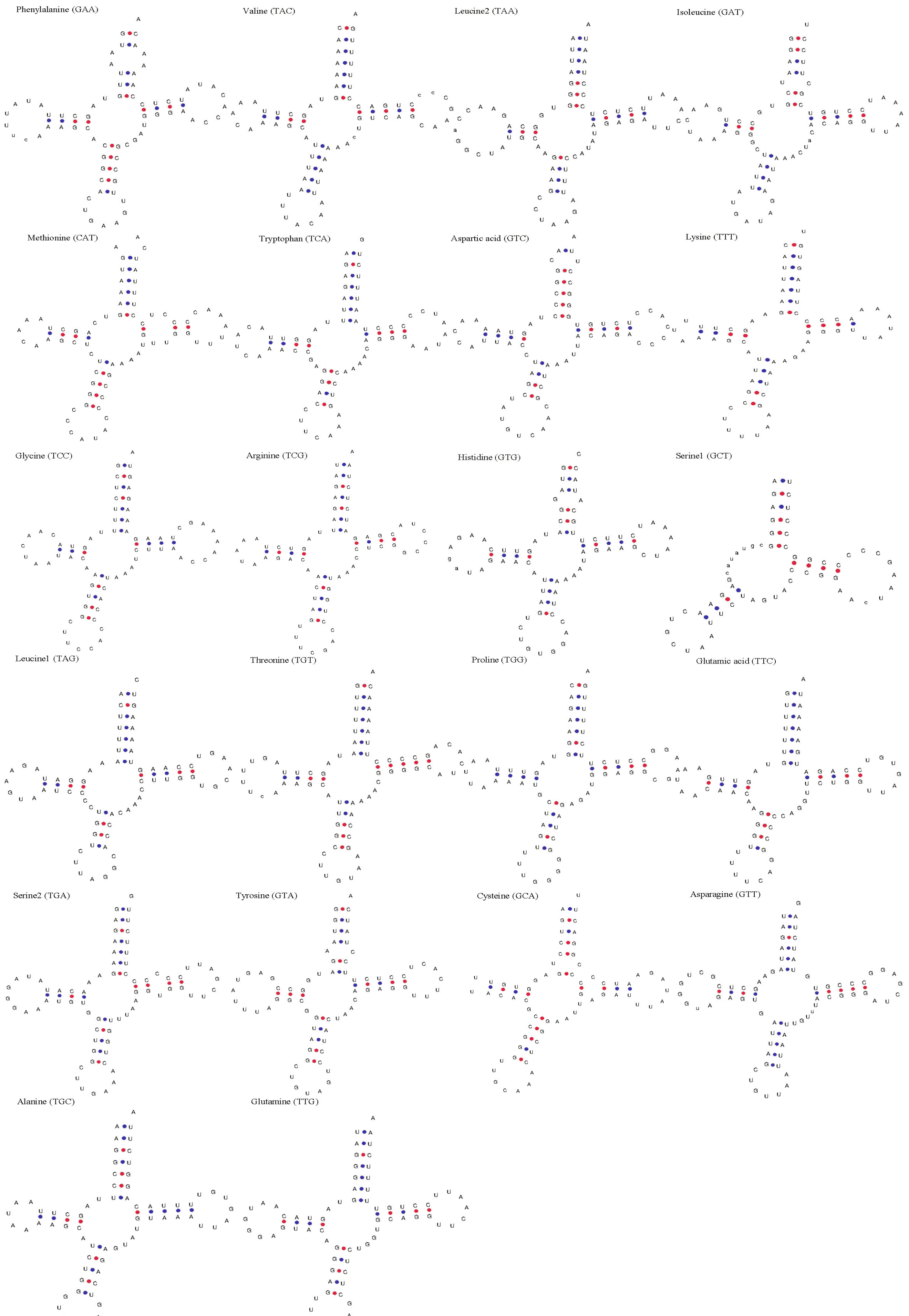
Phenylalanine (GAA)
Valine (TAC)
Leucine2 (TAA)
Isoleucine (GAT)
Methionine (CAT)
Tryptophan (TCA)
Aspartic acid (GTC)
Lysine (TTT)
Glycine (TCC)
Arginine (TCG)
Histidine (GTG)
Serine1 (GCT)
Leucine1 (TAG)
Threonine (TGT)
Proline (TGG)
Glutamic acid (TTC)
Serine2 (TGA)
Tyrosine (GTA)
Cysteine (GCA)
Asparagine (GTT)
Alanine (TGC)
Glutamine (TTG)

MTX

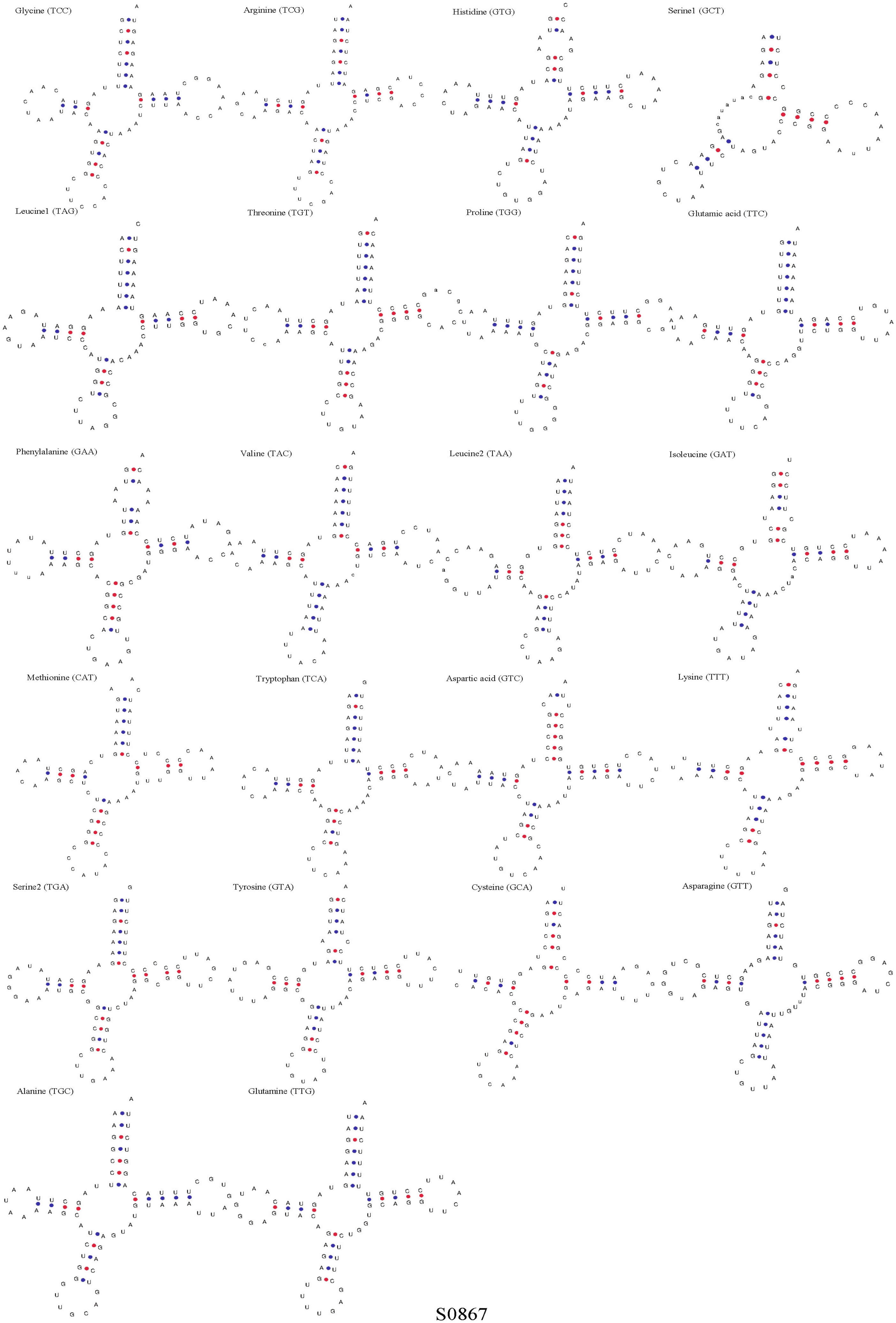
Glycine (TCC)
Arginine (TCG)
Histidine (GTG)
Serine1 (GCT)
Leucine1 (TAG)
Threonine (TGT)
Proline (TGG)
Glutamic acid (TTC)
Phenylalanine (GAA)
Valine (TAC)
Leucine2 (TAA)
Isoleucine (GAT)
Methionine (CAT)
Tryptophan (TCA)
Aspartic acid (GTC)
Lysine (TTT)
Serine2 (TGA)
Tyrosine (GTA)
Cysteine (GCA)
Asparagine (GTT)
Alanine (TGC)
Glutamine (TTG)

S0867

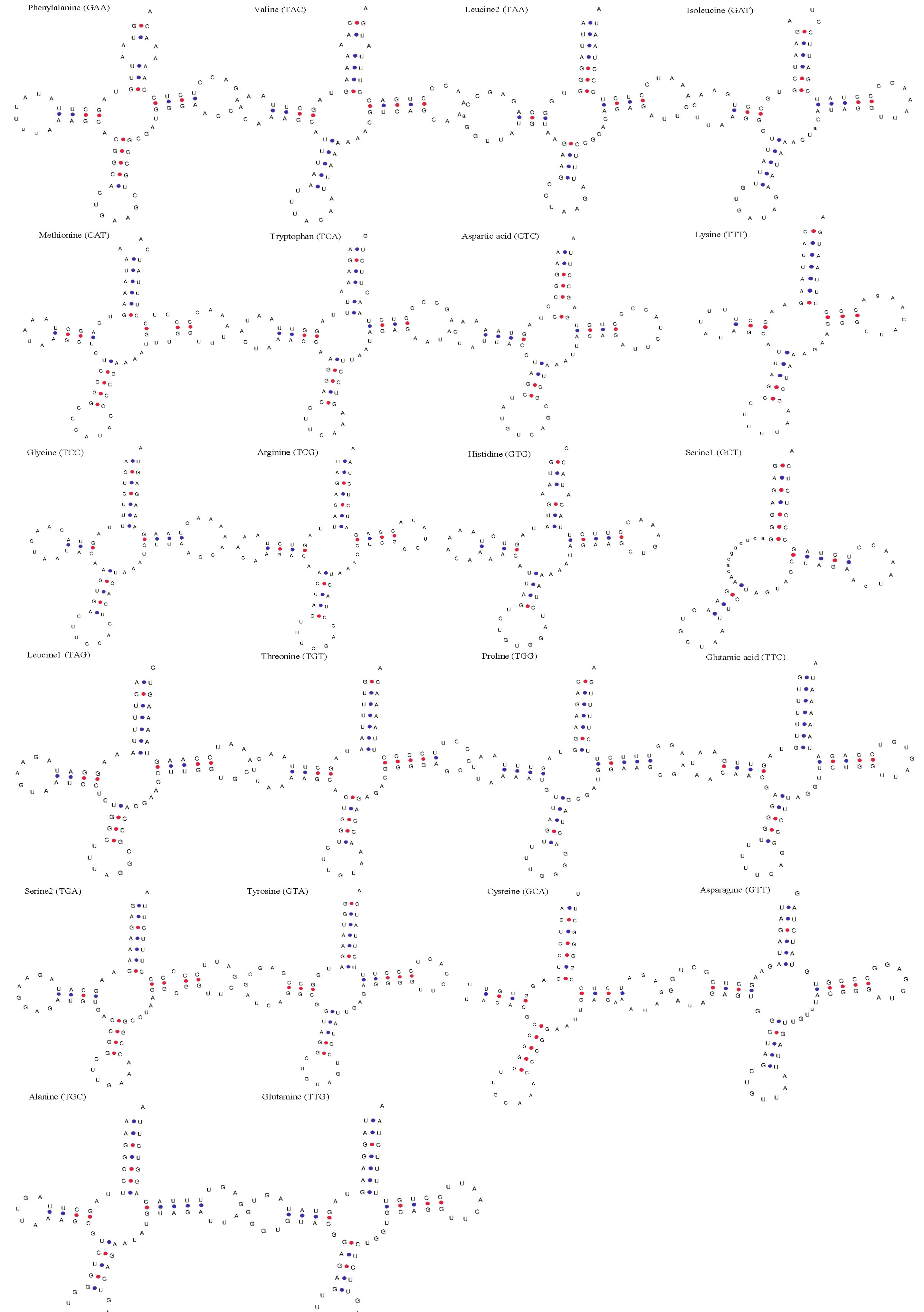
Phenylalanine (GAA)
Valine (TAC)
Leucine2 (TAA)
Isoleucine (GAT)
Methionine (CAT)
Tryptophan (TCA)
Aspartic acid (GTC)
Lysine (TTT)
Glycine (TCC)
Arginine (TCG)
Histidine (GTG)
Serine1 (GCT)
Leucine1 (TAG)
Threonine (TGT)
Proline (TGG)
Glutamic acid (TTC)
Serine2 (TGA)
Tyrosine (GTA)
Cysteine (GCA)
Asparagine (GTT)
Alanine (TGC)
Glutamine (TTG)

TW
