# Supplementary material for: Complete Mitochondrial Genome Sequencing of Asian Glass Lizards (Anguidae: Dopasia): Comparative Analysis With Limbless Anguids and New Insights Into the Adaptive Evolution of Protein‐Coding Genes
Source: Ecol Evol. 2025 Dec 25;15(12):e72811. doi: 10.1002/ece3.72811 (PMC12740153; doi:10.1002/ece3.72811)
Supplement: Supplementary file 5 — Table S1: Codon and anticodon usage for eight complete mitogenomes of Dopasia gracilis (B5008, G1942, MTX, GZ18001, GD04, S0867) and D. harti (TW, CB). [file ECE3-15-e72811-s001.docx]

**Table S1** Codon and anticodon usage for eight complete mitogenomes of *Dopasia gracilis* (B5008, G1942, MTX, GZ18001, GD04, S0867) and *D. harti* (TW, CB).

1. GZ18001

| Gene | Position | | Intergenic  nucleotide | Length (bp) | Codon |  | Anticodon | Strand |
| --- | --- | --- | --- | --- | --- | --- | --- | --- |
|  | From | To |  |  | Start | Stop |  |  |
| tRNA-Phe | 1 | 71 | 0 | 71 |  |  | GAA | H |
| 12S rRNA | 72 | 952 | 0 | 881 |  |  |  | H |
| tRNA-Val | 949 | 1016 | ‒4 | 68 |  |  | TAC | H |
| 16S rRNA | 1017 | 2572 | 0 | 1556 |  |  |  | H |
| tRNA-Leu2 | 2575 | 2647 | 2 | 73 |  |  | TAA | H |
| ND1 | 2649 | 3614 | 1 | 966 | ATG | TAA |  | H |
| tRNA-Ile | 3625 | 3694 | 10 | 70 |  |  | GAT | H |
| tRNA-Gln | 3694 | 3764 | ‒1 | 71 |  |  | TTG | L |
| tRNA-Met | 3764 | 3832 | ‒1 | 69 |  |  | CAT | H |
| ND2 | 3833 | 4870 | 0 | 1038 | ATG | TAG |  | H |
| tRNA-Trp | 4874 | 4941 | 3 | 68 |  |  | TCA | H |
| tRNA-Ala | 4944 | 5012 | 2 | 69 |  |  | TGC | L |
| tRNA-Asn | 5014 | 5086 | 1 | 73 |  |  | GTT | L |
| tRNA-Cys | 5112 | 5177 | 22 | 66 |  |  | GCA | L |
| tRNA-Tyr | 5179 | 5249 | 1 | 71 |  |  | GTA | L |
| COXⅠ | 5251 | 6798 | 1 | 1548 | GTG | TAG |  | H |
| tRNA-Ser2 | 6806 | 6877 | 7 | 72 |  |  | TGA | L |
| tRNA-Asp | 6881 | 6949 | 3 | 69 |  |  | GTC | H |
| COXⅡ | 6950 | 7637 | 0 | 688 | ATG | T-- |  | H |
| tRNA-Lys | 7638 | 7701 | 0 | 64 |  |  | TTT | H |
| ATP8 | 7703 | 7870 | 1 | 168 | ATG | TAA |  | H |
| ATP6 | 7849 | 8544 | ‒22 | 696 | ATG | TAA |  | H |
| COXⅢ | 8544 | 9327 | ‒1 | 784 | ATG | T-- |  | H |
| tRNA-Gly | 9328 | 9396 | 0 | 69 |  |  | TCC | H |
| ND3 | 9397 | 9742 | 0 | 346 | ATG | T-- |  | H |
| tRNA-Arg | 9743 | 9808 | 0 | 66 |  |  | TCG | H |
| ND4L | 9809 | 10,105 | 0 | 297 | ATG | TAA |  | H |
| ND4 | 10,099 | 11,479 | -7 | 1381 | ATG | T-- |  | H |
| tRNA-His | 11,480 | 11,549 | 0 | 70 |  |  | GTG | H |
| tRNA-Ser1 | 11,550 | 11,610 | 0 | 61 |  |  | GCT | H |
| tRNA-Leu1 | 11,610 | 11,680 | -1 | 71 |  |  | TAG | H |
| ND5 | 11,682 | 13,505 | 1 | 1824 | ATG | TAA |  | H |
| ND6 | 13,501 | 14,022 | ‒5 | 522 | ATG | AGG |  | L |
| tRNA-Glu | 14,023 | 14,091 | 0 | 69 |  |  | TTC | L |
| Cyt *b* | 14,095 | 15,231 | 3 | 1137 | ATG | TAG |  | H |
| tRNA-Thr | 15,231 | 15,301 | ‒1 | 71 |  |  | TGT | H |
| tRNA-Pro | 15,302 | 15,369 | 0 | 68 |  |  | TGG | L |
| CR | 15,370 | 15,388 | 0 | 19 |  |  |  | H |

(2) G1942

| Gene | Position | | Intergenic  nucleotide | Length (bp) | Codon |  | Anticodon | Strand |
| --- | --- | --- | --- | --- | --- | --- | --- | --- |
|  | From | To |  |  | Start | Stop |  |  |
| tRNA-Phe | 1 | 71 | 0 | 71 |  |  | GAA | H |
| 12S rRNA | 72 | 1023 | 0 | 952 |  |  |  | H |
| tRNA-Val | 1020 | 1087 | ‒4 | 68 |  |  | TAC | H |
| 16S rRNA | 1118 | 2645 | 0 | 1528 |  |  |  | H |
| tRNA-Leu2 | 2648 | 2720 | 2 | 73 |  |  | TAA | H |
| ND1 | 2722 | 3687 | 1 | 966 | ATG | TAA |  | H |
| tRNA-Ile | 3698 | 3767 | 10 | 70 |  |  | GAT | H |
| tRNA-Gln | 3767 | 3837 | ‒1 | 71 |  |  | TTG | L |
| tRNA-Met | 3837 | 3905 | ‒1 | 69 |  |  | CAT | H |
| ND2 | 3906 | 4943 | 0 | 1038 | ATG | TAG |  | H |
| tRNA-Trp | 4947 | 5014 | 3 | 68 |  |  | TCA | H |
| tRNA-Ala | 5017 | 5085 | 2 | 69 |  |  | TGC | L |
| tRNA-Asn | 5087 | 5159 | 1 | 73 |  |  | GTT | L |
| tRNA-Cys | 5185 | 5250 | 25 | 66 |  |  | GCA | L |
| tRNA-Tyr | 5252 | 5322 | 1 | 71 |  |  | GTA | L |
| COXⅠ | 5324 | 6871 | 1 | 1548 | GTG | TAA |  | H |
| tRNA-Ser2 | 6879 | 6950 | 7 | 72 |  |  | TGA | L |
| tRNA-Asp | 6954 | 7022 | 3 | 69 |  |  | GTC | H |
| COXⅡ | 7023 | 7710 | 0 | 688 | ATG | T-- |  | H |
| tRNA-Lys | 7711 | 7774 | 0 | 64 |  |  | TTT | H |
| ATP8 | 7776 | 7943 | 1 | 168 | ATG | TAA |  | H |
| ATP6 | 7922 | 8617 | -22 | 696 | ATG | TAA |  | H |
| COXⅢ | 8617 | 9400 | -1 | 784 | ATG | T-- |  | H |
| tRNA-Gly | 9401 | 9469 | 0 | 69 |  |  | TCC | H |
| ND3 | 9470 | 9815 | 0 | 346 | ATG | T-- |  | H |
| tRNA-Arg | 9816 | 9881 | 0 | 66 |  |  | TCG | H |
| ND4L | 9882 | 10,178 | 0 | 297 | ATG | TAA |  | H |
| ND4 | 10,172 | 11,552 | ‒7 | 1381 | ATG | T-- |  | H |
| tRNA-His | 11,553 | 11,622 | 0 | 70 |  |  | GTG | H |
| tRNA-Ser1 | 11,623 | 11,683 | 0 | 61 |  |  | GCT | H |
| tRNA-Leu1 | 11,683 | 11,753 | ‒1 | 71 |  |  | TAG | H |
| ND5 | 11,755 | 13,578 | 1 | 1824 | ATG | TAA |  | H |
| ND6 | 13,574 | 14,095 | ‒5 | 522 | ATG | AGG |  | L |
| tRNA-Glu | 14,096 | 14,164 | 0 | 69 |  |  | TTC | L |
| Cyt *b* | 14,168 | 15,303 | 3 | 1136 | ATG | TA- |  | H |
| tRNA-Thr | 15,304 | 15,374 | 0 | 71 |  |  | TGT | H |
| tRNA-Pro | 15,375 | 15,442 | 0 | 68 |  |  | TGG | L |
| CR | 15,443 | 16,923 | 0 | 1481 |  |  |  | H |

(3) MTX

| Gene | Position | | Intergenic  nucleotide | Length (bp) | Codon |  | Anticodon | Strand |
| --- | --- | --- | --- | --- | --- | --- | --- | --- |
|  | From | To |  |  | Start | Stop |  |  |
| tRNA-Phe | 1 | 71 | 0 | 71 |  |  | GAA | H |
| 12S rRNA | 72 | 1019 | 0 | 948 |  |  |  | H |
| tRNA-Val | 1016 | 1084 | ‒4 | 69 |  |  | TAC | H |
| 16S rRNA | 1085 | 2641 | 0 | 1557 |  |  |  | H |
| tRNA-Leu2 | 2644 | 2716 | 2 | 73 |  |  | TAA | H |
| ND1 | 2718 | 3683 | 1 | 966 | ATG | TAA |  | H |
| tRNA-Ile | 3694 | 3763 | 10 | 70 |  |  | GAT | H |
| tRNA-Gln | 3763 | 3833 | ‒1 | 71 |  |  | TTG | L |
| tRNA-Met | 3833 | 3901 | ‒1 | 69 |  |  | CAT | H |
| ND2 | 3902 | 4939 | 0 | 1038 | ATG | TAG |  | H |
| tRNA-Trp | 4943 | 5011 | 3 | 69 |  |  | TCA | H |
| tRNA-Ala | 5014 | 5082 | 2 | 69 |  |  | TGC | L |
| tRNA-Asn | 5084 | 5156 | 1 | 73 |  |  | GTT | L |
| tRNA-Cys | 5182 | 5247 | 25 | 66 |  |  | GCA | L |
| tRNA-Tyr | 5249 | 5319 | 1 | 71 |  |  | GTA | L |
| COXⅠ | 5321 | 6868 | 1 | 1548 | GTG | TAG |  | H |
| tRNA-Ser2 | 6876 | 6947 | 7 | 72 |  |  | TGA | L |
| tRNA-Asp | 6951 | 7019 | 3 | 69 |  |  | GTC | H |
| COXⅡ | 7020 | 7707 | 0 | 688 | ATG | TAT |  | H |
| tRNA-Lys | 7708 | 7771 | 0 | 64 |  |  | TTT | H |
| ATP8 | 7773 | 7940 | 1 | 168 | ATG | TAA |  | H |
| ATP6 | 7919 | 8614 | ‒22 | 696 | ATG | TAA |  | H |
| COXⅢ | 8614 | 9397 | ‒1 | 784 | ATG | T-- |  | H |
| tRNA-Gly | 9398 | 9466 | 0 | 69 |  |  | TCC | H |
| ND3 | 9467 | 9812 | 0 | 346 | ATG | T-- |  | H |
| tRNA-Arg | 9813 | 9878 | 0 | 66 |  |  | TCG | H |
| ND4L | 9879 | 10,175 | 0 | 297 | ATG | TAA |  | H |
| ND4 | 10,169 | 11,549 | ‒7 | 1381 | ATG | T-- |  | H |
| tRNA-His | 11,550 | 11,621 | 0 | 72 |  |  | GTG | H |
| tRNA-Ser1 | 11,622 | 11,682 | 0 | 61 |  |  | GCT | H |
| tRNA-Leu1 | 11,682 | 11,752 | ‒1 | 71 |  |  | TAG | H |
| ND5 | 11,754 | 13,577 | 1 | 1824 | ATG | TAA |  | H |
| ND6 | 13,573 | 14,094 | ‒5 | 522 | ATG | AGG |  | L |
| tRNA-Glu | 14,095 | 14,163 | 0 | 69 |  |  | TTC | L |
| Cyt *b* | 14,167 | 15,302 | 3 | 1136 | ATG | TA- |  | H |
| tRNA-Thr | 15,303 | 15,373 | 0 | 71 |  |  | TGT | H |
| tRNA-Pro | 15,374 | 15,441 | 0 | 68 |  |  | TGG | L |
| CR | 15,442 | 16,447 | 0 | 358 |  |  |  | H |

(4) S0867

| Gene | Position | | Intergenic  nucleotide | Length (bp) | Codon |  | Anticodon | Strand |
| --- | --- | --- | --- | --- | --- | --- | --- | --- |
|  | From | To |  |  | Start | Stop |  |  |
| tRNA-Phe | 1 | 71 | 0 | 71 |  |  | GAA | H |
| 12S rRNA | 72 | 1023 | 0 | 952 |  |  |  | H |
| tRNA-Val | 1020 | 1087 | ‒4 | 68 |  |  | TAC | H |
| 16S rRNA | 1119 | 2646 | 31 | 1528 |  |  |  | H |
| tRNA-Leu2 | 2649 | 2721 | 2 | 73 |  |  | TAA | H |
| ND1 | 2723 | 3688 | 1 | 966 | ATG | TAA |  | H |
| tRNA-Ile | 3699 | 3768 | 10 | 70 |  |  | GAT | H |
| tRNA-Gln | 3768 | 3838 | ‒1 | 71 |  |  | TTG | L |
| tRNA-Met | 3838 | 3905 | ‒1 | 68 |  |  | CAT | H |
| ND2 | 3906 | 4943 | 0 | 1038 | ATG | TAG |  | H |
| tRNA-Trp | 4947 | 5014 | 3 | 68 |  |  | TCA | H |
| tRNA-Ala | 5017 | 5085 | 2 | 69 |  |  | TGC | L |
| tRNA-Asn | 5087 | 5159 | 1 | 73 |  |  | GTT | L |
| tRNA-Cys | 5185 | 5250 | 25 | 66 |  |  | GCA | L |
| tRNA-Tyr | 5252 | 5322 | 1 | 71 |  |  | GTA | L |
| COXⅠ | 5324 | 6871 | 1 | 1548 | GTG | TAA |  | H |
| tRNA-Ser2 | 6879 | 6950 | 7 | 72 |  |  | TGA | L |
| tRNA-Asp | 6954 | 7022 | 3 | 69 |  |  | GTC | H |
| COXⅡ | 7023 | 7710 | 0 | 688 | ATG | TAT |  | H |
| tRNA-Lys | 7711 | 7774 | 0 | 64 |  |  | TTT | H |
| ATP8 | 7776 | 7943 | 1 | 168 | ATG | TAA |  | H |
| ATP6 | 7922 | 8617 | ‒22 | 696 | ATG | TAA |  | H |
| COXⅢ | 8617 | 9400 | ‒1 | 784 | ATG | T-- |  | H |
| tRNA-Gly | 9401 | 9469 | 0 | 69 |  |  | TCC | H |
| ND3 | 9470 | 9815 | 0 | 346 | ATG | T-- |  | H |
| tRNA-Arg | 9816 | 9881 | 0 | 66 |  |  | TCG | H |
| ND4L | 9882 | 10,178 | 0 | 297 | ATG | TAA |  | H |
| ND4 | 10,172 | 11,552 | ‒7 | 1381 | ATG | T-- |  | H |
| tRNA-His | 11,553 | 11,622 | 0 | 70 |  |  | GTG | H |
| tRNA-Ser1 | 11,623 | 11,683 | 0 | 61 |  |  | GCT | H |
| tRNA-Leu1 | 11,683 | 11,753 | ‒1 | 71 |  |  | TAG | H |
| ND5 | 11,755 | 13,578 | 1 | 1824 | ATG | TAA |  | H |
| ND6 | 13,574 | 14,095 | ‒5 | 522 | ATG | AGG |  | L |
| tRNA-Glu | 14,096 | 14,164 | 0 | 69 |  |  | TTC | L |
| Cyt *b* | 14,168 | 15,303 | 3 | 1136 | ATG | TA- |  | H |
| tRNA-Thr | 15,304 | 15,374 | 0 | 71 |  |  | TGT | H |
| tRNA-Pro | 15,375 | 15,442 | 0 | 68 |  |  | TGG | L |
| CR | 15,443 | 16,966 | 0 | 1524 |  |  |  | H |

(5) TW

| Gene | Position | | Intergenic  nucleotide | Length (bp) | Codon |  | Anticodon | Strand |
| --- | --- | --- | --- | --- | --- | --- | --- | --- |
|  | From | To |  |  | Start | Stop |  |  |
| tRNA-Phe | 1 | 71 | 0 | 71 |  |  | GAA | H |
| 12S rRNA | 72 | 1019 | 0 | 948 |  |  |  | H |
| tRNA-Val | 1018 | 1086 | ‒2 | 69 |  |  | TAC | H |
| 16S rRNA | 1087 | 2635 | 0 | 1549 |  |  |  | H |
| tRNA-Leu2 | 2638 | 2710 | 2 | 73 |  |  | TAA | H |
| ND1 | 2712 | 3677 | 1 | 966 | ATG | TAA |  | H |
| tRNA-Ile | 3687 | 3756 | 9 | 70 |  |  | GAT | H |
| tRNA-Gln | 3756 | 3826 | ‒1 | 71 |  |  | TTG | L |
| tRNA-Met | 3826 | 3894 | ‒1 | 69 |  |  | CAT | H |
| ND2 | 3895 | 4932 | 0 | 1038 | ATG | TAG |  | H |
| tRNA-Trp | 4936 | 5006 | 3 | 71 |  |  | TCA | H |
| tRNA-Ala | 5009 | 5077 | 2 | 69 |  |  | TGC | L |
| tRNA-Asn | 5079 | 5151 | 1 | 73 |  |  | GTT | L |
| tRNA-Cys | 5177 | 5241 | 25 | 65 |  |  | GCA | L |
| tRNA-Tyr | 5251 | 5321 | 9 | 71 |  |  | GTA | L |
| COXⅠ | 5323 | 6870 | 1 | 1548 | GTG | TAA |  | H |
| tRNA-Ser2 | 6880 | 6951 | 9 | 72 |  |  | TGA | L |
| tRNA-Asp | 6955 | 7023 | 3 | 69 |  |  | GTC | H |
| COXⅡ | 7024 | 7711 | 0 | 688 | ATG | T-- |  | H |
| tRNA-Lys | 7712 | 7775 | 0 | 64 |  |  | TTT | H |
| ATP8 | 7776 | 7943 | 0 | 168 | ATG | TAA |  | H |
| ATP6 | 7922 | 8617 | ‒22 | 696 | ATG | TAA |  | H |
| COXⅢ | 8617 | 9400 | ‒1 | 784 | ATG | T-- |  | H |
| tRNA-Gly | 9401 | 9469 | 0 | 69 |  |  | TCC | H |
| ND3 | 9470 | 9815 | 0 | 346 | ATG | T-- |  | H |
| tRNA-Arg | 9816 | 9881 | 0 | 66 |  |  | TCG | H |
| ND4L | 9882 | 10,178 | 0 | 297 | ATG | TAA |  | H |
| ND4 | 10,172 | 11,552 | ‒7 | 1381 | ATG | T-- |  | H |
| tRNA-His | 11,553 | 11,622 | 0 | 70 |  |  | GTG | H |
| tRNA-Ser1 | 11,623 | 11,685 | 0 | 63 |  |  | GCT | H |
| tRNA-Leu1 | 11,685 | 11,755 | ‒1 | 71 |  |  | TAG | H |
| ND5 | 11,757 | 13,580 | 1 | 1824 | ATG | TAA |  | H |
| ND6 | 13,576 | 14,097 | ‒5 | 522 | ATG | AGG |  | L |
| tRNA-Glu | 14,098 | 14,166 | 0 | 69 |  |  | TTC | L |
| Cyt *b* | 14,170 | 15,305 | 3 | 1136 | ATG | TA- |  | H |
| tRNA-Thr | 15,306 | 15,375 | 0 | 70 |  |  | TGT | H |
| tRNA-Pro | 15,376 | 15,443 | 0 | 68 |  |  | TGG | L |
| CR | 15,444 | 15,556 | 0 | 113 |  |  |  | H |

(6) B5008

| Gene | Position | | Intergenic  nucleotide | Length (bp) | Codon |  | Anticodon | Strand |
| --- | --- | --- | --- | --- | --- | --- | --- | --- |
|  | From | To |  |  | Start | Stop |  |  |
| tRNA-Phe | 1 | 71 | 0 | 71 |  |  | GAA | H |
| 12S rRNA | 72 | 1019 | 0 | 948 |  |  |  | H |
| tRNA-Val | 1016 | 1084 | ‒4 | 69 |  |  | TAC | H |
| 16S rRNA | 1085 | 2641 | 0 | 1557 |  |  |  | H |
| tRNA-Leu2 | 2644 | 2716 | 2 | 73 |  |  | TAA | H |
| ND1 | 2718 | 3683 | 1 | 966 | ATG | TAA |  | H |
| tRNA-Ile | 3694 | 3763 | 10 | 70 |  |  | GAT | H |
| tRNA-Gln | 3763 | 3833 | ‒1 | 71 |  |  | TTG | L |
| tRNA-Met | 3833 | 3901 | ‒1 | 69 |  |  | CAT | H |
| ND2 | 3923 | 4814 | 21 | 892 | ATG | TAG |  | H |
| tRNA-Trp | 4943 | 5011 | 128 | 69 |  |  | TCA | H |
| tRNA-Ala | 5014 | 5082 | 2 | 69 |  |  | TGC | L |
| tRNA-Asn | 5084 | 5156 | 1 | 73 |  |  | GTT | L |
| tRNA-Cys | 5182 | 5247 | 25 | 66 |  |  | GCA | L |
| tRNA-Tyr | 5249 | 5319 | 1 | 71 |  |  | GTA | L |
| COXⅠ | 5321 | 6868 | 1 | 1548 | GTG | TAG |  | H |
| tRNA-Ser2 | 6876 | 6947 | 7 | 72 |  |  | TGA | L |
| tRNA-Asp | 6951 | 7019 | 3 | 69 |  |  | GTC | H |
| COXⅡ | 7020 | 7707 | 0 | 688 | ATG | T-- |  | H |
| tRNA-Lys | 7708 | 7771 | 0 | 64 |  |  | TTT | H |
| ATP8 | 7773 | 7940 | 1 | 168 | ATG | TAA |  | H |
| ATP6 | 7919 | 8614 | ‒22 | 696 | ATG | TAA |  | H |
| COXⅢ | 8614 | 9397 | ‒1 | 784 | ATG | T-- |  | H |
| tRNA-Gly | 9398 | 9466 | 0 | 69 |  |  | TCC | H |
| ND3 | 9467 | 9812 | 0 | 346 | ATG | T-- |  | H |
| tRNA-Arg | 9813 | 9878 | 0 | 66 |  |  | TCG | H |
| ND4L | 9879 | 10,175 | 0 | 297 | ATG | TAA |  | H |
| ND4 | 10,169 | 11,549 | ‒7 | 1381 | ATG | T-- |  | H |
| tRNA-His | 11,550 | 11,621 | 0 | 72 |  |  | GTG | H |
| tRNA-Ser1 | 11,622 | 11,682 | 0 | 61 |  |  | GCT | H |
| tRNA-Leu1 | 11,682 | 11,752 | ‒1 | 71 |  |  | TAG | H |
| ND5 | 11,754 | 13,577 | 1 | 1824 | ATG | TAA |  | H |
| ND6 | 13,573 | 14,094 | ‒5 | 522 | ATG | AGG |  | L |
| tRNA-Glu | 14,095 | 14,163 | 0 | 69 |  |  | TTC | L |
| Cyt *b* | 14,167 | 15,303 | 3 | 1137 | ATG | TAG |  | H |
| tRNA-Thr | 15,303 | 15,373 | ‒1 | 71 |  |  | TGT | H |
| tRNA-Pro | 15,374 | 15,441 | 0 | 68 |  |  | TGG | L |
| CR | 15,442 | 15,799 | 0 | 358 |  |  |  | H |

(7) CB

| Gene | Position | | Intergenic  nucleotide | Length (bp) | Codon |  | Anticodon | Strand |
| --- | --- | --- | --- | --- | --- | --- | --- | --- |
|  | From | To |  |  | Start | Stop |  |  |
| tRNA-Phe | 1 | 71 | 0 | 71 |  |  | GAA | H |
| 12S rRNA | 72 | 1017 | 0 | 946 |  |  |  | H |
| tRNA-Val | 1016 | 1084 | ‒2 | 69 |  |  | TAC | H |
| 16S rRNA | 1085 | 2637 | 0 | 1552 |  |  |  | H |
| tRNA-Leu2 | 2640 | 2712 | 2 | 73 |  |  | TAA | H |
| ND1 | 2714 | 3679 | 1 | 966 | ATG | TAA |  | H |
| tRNA-Ile | 3689 | 3758 | 9 | 70 |  |  | GAT | H |
| tRNA-Gln | 3758 | 3828 | ‒1 | 71 |  |  | TTG | L |
| tRNA-Met | 3828 | 3896 | ‒1 | 69 |  |  | CAT | H |
| ND2 | 3897 | 4934 | 0 | 1038 | ATG | TAG |  | H |
| tRNA-Trp | 4938 | 5008 | 3 | 71 |  |  | TCA | H |
| tRNA-Ala | 5011 | 5079 | 2 | 69 |  |  | TGC | L |
| tRNA-Asn | 5081 | 5153 | 1 | 73 |  |  | GTT | L |
| tRNA-Cys | 5179 | 5243 | 25 | 65 |  |  | GCA | L |
| tRNA-Tyr | 5251 | 5321 | 7 | 71 |  |  | GTA | L |
| COXⅠ | 5323 | 6870 | 1 | 1548 | GTG | TAA |  | H |
| tRNA-Ser2 | 6882 | 6953 | 11 | 72 |  |  | TGA | L |
| tRNA-Asp | 6957 | 7025 | 3 | 69 |  |  | GTC | H |
| COXⅡ | 7026 | 7713 | 0 | 688 | ATG | T-- |  | H |
| tRNA-Lys | 7714 | 7777 | 0 | 64 |  |  | TTT | H |
| ATP8 | 7778 | 7945 | 0 | 168 | ATG | TAA |  | H |
| ATP6 | 7924 | 8619 | ‒22 | 696 | ATG | TAA |  | H |
| COXⅢ | 8616 | 9402 | ‒4 | 787 | ATA | T-- |  | H |
| tRNA-Gly | 9403 | 9470 | 0 | 68 |  |  | TCC | H |
| ND3 | 9471 | 9816 | 0 | 346 | ATG | T-- |  | H |
| tRNA-Arg | 9817 | 9882 | 0 | 66 |  |  | TCG | H |
| ND4L | 9883 | 10,179 | 0 | 297 | ATG | TAA |  | H |
| ND4 | 10,173 | 11,553 | ‒7 | 1381 | ATG | T-- |  | H |
| tRNA-His | 11,554 | 11,623 | 0 | 70 |  |  | GTG | H |
| tRNA-Ser1 | 11,624 | 11,687 | 0 | 64 |  |  | GCT | H |
| tRNA-Leu1 | 11,687 | 11,757 | ‒1 | 71 |  |  | TAG | H |
| ND5 | 11,759 | 13,582 | 1 | 1824 | ATG | TAA |  | H |
| ND6 | 13,578 | 14,099 | ‒5 | 522 | ATG | AGG |  | L |
| tRNA-Glu | 14,100 | 14,168 | 0 | 69 |  |  | TTC | L |
| Cyt *b* | 14,172 | 15,307 | 3 | 1136 | ATG | TA- |  | H |
| tRNA-Thr | 15,308 | 15,376 | 0 | 69 |  |  | TGT | H |
| tRNA-Pro | 15,377 | 15,444 | 0 | 68 |  |  | TGG | L |
| CR | 15,445 | 17,000 | 0 | 1556 |  |  |  | H |

(8) GD04

| Gene | Position | | Intergenic  nucleotide | Length (bp) | Codon |  | Anticodon | Strand |
| --- | --- | --- | --- | --- | --- | --- | --- | --- |
|  | From | To |  |  | Start | Stop |  |  |
| tRNA-Phe | 1 | 70 | 0 | 70 |  |  | GAA | H |
| 12S rRNA | 71 | 1013 | 0 | 943 |  |  |  | H |
| tRNA-Val | 1014 | 1082 | 0 | 69 |  |  | TAC | H |
| 16S rRNA | 1083 | 2634 | 0 | 1552 |  |  |  | H |
| tRNA-Leu2 | 2635 | 2707 | 0 | 73 |  |  | TAA | H |
| ND1 | 2709 | 3674 | 1 | 966 | ATG | TAA |  | H |
| tRNA-Ile | 3684 | 3753 | 9 | 70 |  |  | GAT | H |
| tRNA-Gln | 3753 | 3823 | ‒1 | 71 |  |  | TTG | L |
| tRNA-Met | 3823 | 3891 | ‒1 | 69 |  |  | CAT | H |
| ND2 | 3892 | 4929 | 0 | 1038 | ATG | TAG |  | H |
| tRNA-Trp | 4933 | 5003 | 3 | 71 |  |  | TCA | H |
| tRNA-Ala | 5006 | 5074 | 2 | 69 |  |  | TGC | L |
| tRNA-Asn | 5076 | 5148 | 1 | 73 |  |  | GTT | L |
| tRNA-Cys | 5174 | 5238 | 25 | 65 |  |  | GCA | L |
| tRNA-Tyr | 5246 | 5316 | 7 | 71 |  |  | GTA | L |
| COXⅠ | 5318 | 6865 | 1 | 1548 | GTG | TAA |  | H |
| tRNA-Ser2 | 6877 | 6948 | 11 | 72 |  |  | TGA | L |
| tRNA-Asp | 6952 | 7020 | 3 | 69 |  |  | GTC | H |
| COXⅡ | 7021 | 7708 | 0 | 688 | ATG | T-- |  | H |
| tRNA-Lys | 7709 | 7772 | 0 | 64 |  |  | TTT | H |
| ATP8 | 7773 | 7940 | 0 | 168 | ATG | TAA |  | H |
| ATP6 | 7919 | 8614 | ‒22 | 696 | ATG | TAA |  | H |
| COXⅢ | 8614 | 9397 | ‒1 | 784 | ATG | T-- |  | H |
| tRNA-Gly | 9398 | 9466 | 0 | 69 |  |  | TCC | H |
| ND3 | 9467 | 9812 | 0 | 346 | ATG | T-- |  | H |
| tRNA-Arg | 9813 | 9878 | 0 | 66 |  |  | TCG | H |
| ND4L | 9879 | 10,175 | 0 | 297 | ATG | TAA |  | H |
| ND4 | 10,169 | 11,549 | ‒7 | 1381 | ATG | T-- |  | H |
| tRNA-His | 11,550 | 11,619 | 0 | 70 |  |  | GTG | H |
| tRNA-Ser1 | 11,620 | 11,683 | 0 | 64 |  |  | GCT | H |
| tRNA-Leu1 | 11,683 | 11,753 | ‒1 | 71 |  |  | TAG | H |
| ND5 | 11,755 | 13,578 | 1 | 1824 | ATG | TAA |  | H |
| ND6 | 13,618 | 14,095 | 39 | 478 | ATG | G-- |  | L |
| tRNA-Glu | 14,096 | 14,164 | 0 | 69 |  |  | TTC | L |
| Cyt *b* | 14,168 | 15,303 | 3 | 1136 | ATG | TA- |  | H |
| tRNA-Thr | 15,304 | 15,373 | 0 | 70 |  |  | TGT | H |
| tRNA-Pro | 15,374 | 15,441 | 0 | 68 |  |  | TGG | L |
| CR | 15,442 | 17,070 | 0 | 1629 |  |  |  | H |
