# Supplementary material for: Complete Mitochondrial Genome Sequencing of Asian Glass Lizards (Anguidae: Dopasia): Comparative Analysis With Limbless Anguids and New Insights Into the Adaptive Evolution of Protein‐Coding Genes
Source: Ecol Evol. 2025 Dec 25;15(12):e72811. doi: 10.1002/ece3.72811 (PMC12740153; doi:10.1002/ece3.72811)
Supplement: Supplementary file 6 — Table S2: Base composition of eight Dopasia mitogenomes. [file ECE3-15-e72811-s004.docx]

Table S2. Base percent composition of complete mitogenome sequences of 8 individuals in *Dopasia* generated in MEGA 7.0.26.

| Gene | | *Dopasia gracilis* B5008 | *Dopasia gracilis* G1942 | *Dopasia gracilis* MTX | *Dopasia gracilis* GZ18001 | *Dopasia gracilis* GD04 | *Dopasia gracilis* S0867 | *Dopasia harti* TW | *Dopasia harti* CB |
| --- | --- | --- | --- | --- | --- | --- | --- | --- | --- |
| ATP6 | T | 25.7 | 27.0 | 25.7 | 27.0 | 25.3 | 27.2 | 25.1 | 24.4 |
|  | C | 33.5 | 32.8 | 33.5 | 32.8 | 33.2 | 32.6 | 33.3 | 33.9 |
|  | A | 29.3 | 29.5 | 29.3 | 29.5 | 29.7 | 29.0 | 31.2 | 30.9 |
|  | G | 11.5 | 10.8 | 11.5 | 10.8 | 11.8 | 11.2 | 10.3 | 10.8 |
|  | Total | 696 | 696 | 696 | 696 | 696 | 696 | 696 | 696 |
| ATP8 | T | 20.2 | 20.8 | 20.2 | 20.8 | 22.0 | 20.2 | 20.2 | 22.0 |
|  | C | 37.5 | 37.5 | 37.5 | 37.5 | 33.3 | 38.1 | 35.1 | 33.9 |
|  | A | 32.7 | 31.5 | 32.7 | 31.5 | 37.5 | 31.0 | 36.3 | 36.9 |
|  | G | 9.5 | 10.1 | 9.5 | 10.1 | 7.1 | 10.7 | 8.3 | 7.1 |
|  | Total | 168 | 168 | 168 | 168 | 168 | 168 | 168 | 168 |
| COXⅠ | T | 26.0 | 26.2 | 26.0 | 26.3 | 25.8 | 26.2 | 25.9 | 26.2 |
|  | C | 31.3 | 31.3 | 31.3 | 31.3 | 30.4 | 31.3 | 30.4 | 30.2 |
|  | A | 23.6 | 23.6 | 23.6 | 23.6 | 25.0 | 23.7 | 24.7 | 25.5 |
|  | G | 19.1 | 18.8 | 19.1 | 18.8 | 18.8 | 18.7 | 19.0 | 18.1 |
|  | Total | 1548 | 1548 | 1548 | 1548 | 1548 | 1548 | 1548 | 1548 |
| COXⅡ | T | 23.1 | 23.4 | 23.1 | 23.4 | 23.0 | 23.3 | 22.1 | 22.1 |
|  | C | 30.7 | 31.4 | 30.7 | 31.4 | 31.3 | 31.5 | 32.1 | 31.8 |
|  | A | 31.5 | 30.5 | 31.5 | 30.5 | 31.4 | 30.5 | 31.4 | 31.5 |
|  | G | 14.7 | 14.7 | 14.7 | 14.7 | 14.4 | 14.7 | 14.4 | 14.5 |
|  | Total | 688 | 688 | 688 | 688 | 688 | 688 | 688 | 688 |
| COXⅢ | T | 25.1 | 25.5 | 25.1 | 25.5 | 25.0 | 25.5 | 24.7 | 25.4 |
|  | C | 31.1 | 30.9 | 31.1 | 30.9 | 31.0 | 30.9 | 30.9 | 30.7 |
|  | A | 26.3 | 26.4 | 26.1 | 26.4 | 26.7 | 26.1 | 27.6 | 27.6 |
|  | G | 17.5 | 17.2 | 17.6 | 17.2 | 17.3 | 17.5 | 16.8 | 16.3 |
|  | Total | 784 | 784 | 784 | 784 | 784 | 784 | 784 | 787 |
| CYTB | T | 25.2 | 26.8 | 25.3 | 26.8 | 25.9 | 26.6 | 25.4 | 25.6 |
|  | C | 32.4 | 30.9 | 32.4 | 30.9 | 32.3 | 31.2 | 32.5 | 32.6 |
|  | A | 28.1 | 27.8 | 28.1 | 27.8 | 29.7 | 27.8 | 29.5 | 29.9 |
|  | G | 14.3 | 14.4 | 14.3 | 14.5 | 12.1 | 14.4 | 12.7 | 11.9 |
|  | Total | 1137 | 1136 | 1136 | 1137 | 1136 | 1136 | 1136 | 1136 |
| ND1 | T | 27.0 | 26.2 | 27.0 | 26.2 | 24.8 | 26.3 | 25.6 | 26.8 |
|  | C | 29.2 | 29.8 | 29.2 | 29.8 | 31.2 | 29.7 | 30.4 | 30.0 |
|  | A | 29.2 | 28.9 | 29.2 | 28.9 | 29.9 | 28.7 | 29.5 | 28.9 |
|  | G | 14.6 | 15.1 | 14.6 | 15.1 | 14.1 | 15.3 | 14.5 | 14.3 |
|  | Total | 966 | 966 | 966 | 966 | 966 | 966 | 966 | 966 |
| ND2 | T | 23.1 | 23.2 | 23.1 | 23.2 | 21.6 | 23.0 | 21.8 | 22.5 |
|  | C | 33.0 | 33.1 | 33.0 | 33.1 | 33.8 | 33.1 | 32.9 | 33.4 |
|  | A | 32.1 | 32.2 | 32.1 | 32.2 | 33.9 | 32.3 | 34.7 | 33.1 |
|  | G | 11.8 | 11.5 | 11.8 | 11.5 | 10.7 | 11.6 | 10.6 | 10.9 |
|  | Total | 1038 | 1038 | 1038 | 1038 | 1038 | 1038 | 1038 | 1038 |
| ND3 | T | 27.7 | 28.0 | 27.7 | 28.0 | 26.6 | 28.0 | 28.3 | 26.0 |
|  | C | 33.8 | 33.8 | 33.8 | 33.8 | 33.2 | 33.8 | 31.5 | 34.4 |
|  | A | 22.8 | 22.3 | 22.8 | 22.3 | 26.3 | 22.3 | 25.1 | 25.7 |
|  | G | 15.6 | 15.9 | 15.6 | 15.9 | 13.9 | 15.9 | 15.0 | 13.9 |
|  | Total | 346 | 346 | 346 | 346 | 346 | 346 | 346 | 346 |
| ND4 | T | 24.8 | 25.0 | 24.8 | 25.0 | 24.9 | 25.1 | 24.4 | 24.7 |
|  | C | 34.0 | 33.8 | 34.0 | 33.8 | 33.8 | 33.7 | 34.5 | 34.4 |
|  | A | 29.2 | 29.5 | 29.1 | 29.5 | 29.3 | 29.6 | 29.5 | 28.7 |
|  | G | 12.1 | 11.7 | 12.1 | 11.7 | 11.9 | 11.6 | 11.5 | 12.2 |
|  | Total | 1381 | 1381 | 1380 | 1381 | 1381 | 1381 | 1381 | 1381 |
| ND4L | T | 26.6 | 26.6 | 26.6 | 26.6 | 28.3 | 26.6 | 26.9 | 27.6 |
|  | C | 33.0 | 32.7 | 33.0 | 32.7 | 32.0 | 32.7 | 32.3 | 33.0 |
|  | A | 26.6 | 26.9 | 26.6 | 26.9 | 25.6 | 26.9 | 28.3 | 24.6 |
|  | G | 13.8 | 13.8 | 13.8 | 13.8 | 14.1 | 13.8 | 12.5 | 14.8 |
|  | Total | 297 | 297 | 297 | 297 | 297 | 297 | 297 | 297 |
| ND5 | T | 23.4 | 23.7 | 23.4 | 23.7 | 23.5 | 23.7 | 22.8 | 23.6 |
|  | C | 32.2 | 32.0 | 32.2 | 32.0 | 31.5 | 32.0 | 32.3 | 31.6 |
|  | A | 31.4 | 30.9 | 31.3 | 30.9 | 31.6 | 31.1 | 32.0 | 31.5 |
|  | G | 13.0 | 13.3 | 13.0 | 13.3 | 13.4 | 13.2 | 12.9 | 13.3 |
|  | Total | 1824 | 1824 | 1824 | 1824 | 1824 | 1824 | 1824 | 1824 |
| ND6 | T | 43.3 | 44.1 | 43.3 | 44.1 | 44.6 | 44.1 | 45.8 | 45.2 |
|  | C | 12.3 | 11.1 | 12.3 | 11.1 | 10.9 | 11.1 | 9.6 | 10.2 |
|  | A | 12.3 | 12.8 | 12.3 | 12.8 | 12.3 | 13.0 | 11.3 | 12.5 |
|  | G | 32.2 | 32.0 | 32.2 | 32.0 | 32.2 | 31.8 | 33.3 | 32.2 |
|  | Total | 522 | 522 | 522 | 522 | 478 | 522 | 522 | 522 |
| CR | T | 23.5 | 26.2 | 17.2 | 27.2 | 24.2 | 26.1 | 24.8 | 26.4 |
|  | C | 33 | 28.8 | 41.6 | 27.7 | 31.4 | 29 | 31.7 | 30.7 |
|  | A | 30.1 | 30.4 | 28 | 31 | 33.1 | 30.5 | 31.6 | 31.2 |
|  | G | 13.5 | 14.5 | 13.3 | 14.1 | 11.3 | 14.4 | 11.9 | 11.7 |
|  | Total | 1777 | 1799 | 1379 | 1406 | 1629 | 1830 | 1626 | 1556 |
|  | %GC | 46.5 | 43.3 | 54.9 | 41.8 | 42.7 | 43.4 | 43.6 | 42.4 |
|  | %AT | 53.6 | 56.6 | 45.2 | 58.2 | 57.3 | 56.6 | 56.4 | 57.6 |
| 12S rRNA | T | 19.4 | 19.9 | 19.4 | 19.9 | 19.7 | 19.6 | 19.6 | 19.2 |
|  | C | 26.9 | 26.9 | 26.9 | 26.9 | 26.6 | 27.1 | 27.2 | 27.1 |
|  | A | 35.3 | 34.8 | 35.3 | 35.0 | 35.7 | 34.9 | 35.8 | 35.6 |
|  | G | 18.4 | 18.5 | 18.4 | 18.2 | 17.9 | 18.4 | 17.4 | 18.1 |
|  | Total | 948 | 952 | 948 | 881 | 943 | 953 | 949 | 946 |
| 16S rRNA | T | 19.3 | 19.0 | 19.2 | 18.8 | 19.4 | 19.0 | 19.8 | 19.3 |
|  | C | 27.7 | 27.6 | 27.7 | 28.1 | 27.8 | 27.6 | 27.2 | 27.9 |
|  | A | 35.3 | 35.4 | 35.4 | 35.1 | 35.2 | 35.3 | 35.4 | 34.7 |
|  | G | 17.7 | 18.1 | 17.7 | 18.0 | 17.6 | 18.1 | 17.5 | 18.1 |
|  | Total | 1557 | 1528 | 1557 | 1556 | 1552 | 1529 | 1550 | 1553 |
| rRNA | T | 19.3 | 19.4 | 19.3 | 19.3 | 19.6 | 19.3 | 19.7 | 19.3 |
|  | C | 27.3 | 27.2 | 27.3 | 27.5 | 27.2 | 27.4 | 27.2 | 27.5 |
|  | A | 35.3 | 35.1 | 35.4 | 35.1 | 35.5 | 35.1 | 35.6 | 35.2 |
|  | G | 18.0 | 18.3 | 18.0 | 18.1 | 17.8 | 18.2 | 17.5 | 18.1 |
|  | %AT | 54.7 | 54.5 | 54.7 | 54.4 | 55.0 | 54.4 | 55.3 | 54.4 |
|  | %GC | 45.3 | 45.5 | 45.3 | 45.6 | 45.0 | 45.6 | 44.7 | 45.6 |
| tRNA | Total | 1523 | 1519 | 1523 | 1519 | 1523 | 1518 | 1523 | 1522 |
| Complete mitogenome | T | 23.6 | 24.1 | 23.1 | 24.2 | 23.5 | 24.1 | 23.4 | 23.8 |
|  | C | 30.9 | 30.4 | 31.6 | 30.3 | 30.7 | 30.4 | 30.8 | 30.7 |
|  | A | 30.5 | 30.5 | 30.4 | 30.5 | 31.5 | 30.5 | 31.6 | 31.2 |
|  | G | 14.9 | 15.0 | 15.0 | 14.9 | 14.3 | 15.0 | 14.2 | 14.3 |
|  | Total | 1,7218 | 1,7241 | 1,6823 | 1,6775 | 1,7070 | 1,7272 | 1,7069 | 1,7000 |
|  | %GC | 45.876 | 45.380 | 46.560 | 45.272 | 44.985 | 45.432 | 44.965 | 45.024 |
|  | %AT | 54.124 | 54.620 | 53.440 | 54.728 | 55.015 | 54.568 | 55.035 | 54.976 |
|  | GC Skew | -0.348 | -0.339 | -0.357 | -0.340 | -0.363 | -0.339 | -0.369 | -0.364 |
|  | AT Skew | 0.128 | 0.116 | 0.137 | 0.115 | 0.145 | 0.117 | 0.148 | 0.136 |
