# Supplementary material for: Complete Mitochondrial Genome Sequencing of Asian Glass Lizards (Anguidae: Dopasia): Comparative Analysis With Limbless Anguids and New Insights Into the Adaptive Evolution of Protein‐Coding Genes
Source: Ecol Evol. 2025 Dec 25;15(12):e72811. doi: 10.1002/ece3.72811 (PMC12740153; doi:10.1002/ece3.72811)
Supplement: Supplementary file 7 — Table S3: CodeML site model specifications. [file ECE3-15-e72811-s009.docx]

Table S3. Detailed information on the site models used in CodeML

ATP6

| Model | Np | LnL | Estemates of parameters | |  |  | Model  compared | LRT P-value |
| --- | --- | --- | --- | --- | --- | --- | --- | --- |
| M0 | 38 | -4854.016318 | ω: | 0.09903 |  |  |  |  |
| M1a | 39 | -4804.235813 | p: | 0.92080 | 0.07920 |  | M0vs.M1a | 0.000000000 |
|  |  |  | ω: | 0.06387 | 1.00000 |  |  |  |
| M2a | 41 | -4804.235813 | p: | 0.92080 | 0.06571 | 0.01350 | M1avs.M2a | 1.000000000 |
|  |  |  | ω: | 0.06387 | 1.00000 | 1.00000 |  |  |
| M7 | 39 | -4769.935002 | p=0.28008 |  | q=2.24176 |  |  |  |
| M8 | 41 | -4768.661599 | p0=0.99484 | p=0.30026 | q=2.60842 |  | M7vs.M8 | 0.279878416 |
|  |  |  | (p1=0.00516) | ω=1.66125 |  |  |  |  |

ATP8

| Model | Np | LnL | Estemates of parameters | |  |  | Model  compared | LRT P-value |
| --- | --- | --- | --- | --- | --- | --- | --- | --- |
| M0 | 38 | -1385.802605 | ω: | 0.22935 |  |  |  |  |
| M1a | 39 | -1350.177439 | p: | 0.65384 | 0.34616 |  | M0vs.M1a | 0.000000000 |
|  |  |  | ω: | 0.07225 | 1.00000 |  |  |  |
| M2a | 41 | -1350.177439 | p: | 0.65384 | 0.26396 | 0.08220 | M1avs.M2a | 1.000000000 |
|  |  |  | ω: | 0.07225 | 1.00000 | 1.00000 |  |  |
| M7 | 39 | -1338.379380 | p=0.28055 |  | q=0.74338 |  |  |  |
| M8 | 41 | -1338.332429 | p0=0.98585 | p=0.28989 | q=0.81651 |  | M7vs.M8 | 0.954135103 |
|  |  |  | (p1=0.01415) | ω=1.50639 |  |  |  |  |

COXⅠ

| Model | Np | LnL | Estemates of parameters | |  |  | Model  compared | LRT P-value |
| --- | --- | --- | --- | --- | --- | --- | --- | --- |
| M0 | 38 | -8825.657435 | ω: | 0.01784 |  |  |  |  |
| M1a | 39 | -8755.732045 | p: | 0.97709 | 0.02291 |  | M0vs.M1a | 0.000000000 |
|  |  |  | ω: | 0.01018 | 1.00000 |  |  |  |
| M2a | 41 | -8755.732046 | p: | 0.97709 | 0.02291 | 0.00000 | M1avs.M2a | 1.000000000 |
|  |  |  | ω: | 0.01018 | 1.00000 | 38.68911 |  |  |
| M7 | 39 | -8717.401263 | p=0.06942 |  | q=2.35190 |  |  |  |
| M8 | 41 | -8717.406403 | p0=0.99999 | p=0.06942 | q=2.35190 |  | M7vs.M8 | 0.994863239 |
|  |  |  | (p1=0.00001) | ω=23.73444 |  |  |  |  |

COXⅡ

| Model | Np | LnL | Estemates of parameters | |  |  | Model  compared | LRT P-value |
| --- | --- | --- | --- | --- | --- | --- | --- | --- |
| M0 | 38 | -3785.541011 | ω: | 0.04796 |  |  |  |  |
| M1a | 39 | -3772.555079 | p: | 0.95606 | 0.04394 |  | M0vs.M1a | 0.000000346 |
|  |  |  | ω: | 0.03517 | 1.00000 |  |  |  |
| M2a | 41 | -3772.555079 | p: | 0.95606 | 0.04394 | 0.00000 | M1avs.M2a | 1.000000000 |
|  |  |  | ω: | 0.03517 | 1.00000 | 25.35899 |  |  |
| M7 | 39 | -3742.030650 | p=0.23053 |  | q=4.10427 |  |  |  |
| M8 | 41 | -3742.032932 | p0=0.99999 | p=0.23054 | q=4.10433 |  | M7vs.M8 | 0.997702643 |
|  |  |  | (p1=0.00001) | ω=4.18003 |  |  |  |  |

COXⅢ

| Model | Np | LnL | Estemates of parameters | |  |  | Model  compared | LRT P-value |
| --- | --- | --- | --- | --- | --- | --- | --- | --- |
| M0 | 38 | -4734.117923 | ω: | 0.05348 |  |  |  |  |
| M1a | 39 | -4679.941213 | p: | 0.95223 | 0.04777 |  | M0vs.M1a | 0.000000000 |
|  |  |  | ω: | 0.03530 | 1.00000 |  |  |  |
| M2a | 41 | -4679.941234 | p: | 0.95222 | 0.04778 | 0.00000 | M1avs.M2a | 1.000000000 |
|  |  |  | ω: | 0.03530 | 1.00000 | 4.33877 |  |  |
| M7 | 39 | -4639.915160 | p=0.16835 |  | q=2.30540 |  |  |  |
| M8 | 41 | -4639.917757 | p0=0.99999 | p=0.16835 | q=2.30540 |  | M7vs.M8 | 0.997403377 |
|  |  |  | (p1=0.00001) | ω=6.85117 |  |  |  |  |

CYTB

| Model | Np | LnL | Estemates of parameters | |  |  | Model  compared | LRT P-value |
| --- | --- | --- | --- | --- | --- | --- | --- | --- |
| M0 | 38 | -4963.117549 | ω: | 0.05128 |  |  |  |  |
| M1a | 39 | -4887.910740 | p: | 0.93125 | 0.06875 |  | M0vs.M1a | 0.000000000 |
|  |  |  | ω: | 0.02649 | 1.00000 |  |  |  |
| M2a | 41 | -4887.910740 | p: | 0.93125 | 0.06235 | 0.00640 | M1avs.M2a | 1.000000000 |
|  |  |  | ω: | 0.02649 | 1.00000 | 1.00000 |  |  |
| M7 | 39 | -4841.626732 | p=0.13738 |  | q=2.02832 |  |  |  |
| M8 | 41 | -4839.799315 | p0=0.99588 | p=0.14563 | q=2.42762 |  | M7vs.M8 | 0.160831186 |
|  |  |  | (p1=0.00412) | ω=1.38597 |  |  |  |  |

ND1

| Model | Np | LnL | Estemates of parameters | |  |  | Model  compared | LRT P-value |
| --- | --- | --- | --- | --- | --- | --- | --- | --- |
| M0 | 38 | -5967.2716469 | ω: | 0.04864 |  |  |  |  |
| M1a | 39 | -5922.000400 | p: | 0.93342 | 0.06658 |  | M0vs.M1a | 0.000000000 |
|  |  |  | ω: | 0.03018 | 1.00000 |  |  |  |
| M2a | 41 | -5922.000400 | p: | 0.93342 | 0.06658 | 0.00000 | M1avs.M2a | 1.000000000 |
|  |  |  | ω: | 0.03018 | 1.00000 | 24.93845 |  |  |
| M7 | 39 | -5860.961197 | p=0.15410 |  | q=2.46857 |  |  |  |
| M8 | 41 | -5860.962129 | p0=0.99999 | p=0.15411 | q=2.46888 |  | M7vs.M8 | 0.999050451 |
|  |  |  | (p1=0.00001) | ω=1.00000 |  |  |  |  |

ND2

| Model | Np | LnL | Estemates of parameters | |  |  | Model  compared | LRT P-value |
| --- | --- | --- | --- | --- | --- | --- | --- | --- |
| M0 | 38 | -6828.478794 | ω: | 0.07028 |  |  |  |  |
| M1a | 39 | -6753.268644 | p: | 0.94227 | 0.05773 |  | M0vs.M1a | 0.000000000 |
|  |  |  | ω: | 0.04800 | 1.00000 |  |  |  |
| M2a | 41 | -6753.268644 | p: | 0.94227 | 0.05773 | 0.00000 | M1avs.M2a | 1.000000000 |
|  |  |  | ω: | 0.04800 | 1.00000 | 30.11126 |  |  |
| M7 | 39 | -6716.070675 | p=0.29888 |  | q=3.29764 |  |  |  |
| M8 | 41 | -6709.003091 | p0=0.98201 | p=0.38397 | q=5.53248 |  | M7vs.M8 | 0.000852276 |
|  |  |  | (p1=0.01799) | ω=1.10783 |  |  |  |  |

ND3

| Model | Np | LnL | Estemates of parameters | |  |  | Model  compared | LRT P-value |
| --- | --- | --- | --- | --- | --- | --- | --- | --- |
| M0 | 38 | -2271.057723 | ω: | 0.08051 |  |  |  |  |
| M1a | 39 | -2242.361919 | p: | 0.89979 | 0.10021 |  | M0vs.M1a | 0.000000000 |
|  |  |  | ω: | 0.04840 | 1.00000 |  |  |  |
| M2a | 41 | -2242.361902 | p: | 0.89979 | 0.06703 | 0.03319 | M1avs.M2a | 1.000000000 |
|  |  |  | ω: | 0.04840 | 1.00000 | 1.00000 |  |  |
| M7 | 39 | -2217.306640 | p=0.19837 |  | q=1.87493 |  |  |  |
| M8 | 41 | -2217.306981 | p0=0.99999 | p=0.19837 | q=1.87506 |  | M7vs.M8 | 0.999650061 |
|  |  |  | (p1=0.00001) | ω=1.00000 |  |  |  |  |

ND4

| Model | Np | LnL | Estemates of parameters | |  |  | Model  compared | LRT P-value |
| --- | --- | --- | --- | --- | --- | --- | --- | --- |
| M0 | 38 | -8968.650563 | ω: | 0.07577 |  |  |  |  |
| M1a | 39 | -8858.054772 | p: | 0.92269 | 0.07731 |  | M0vs.M1a | 0.000000000 |
|  |  |  | ω: | 0.04671 | 1.00000 |  |  |  |
| M2a | 41 | -8858.054772 | p: | 0.92269 | 0.07731 | 0.00000 | M1avs.M2a | 1.000000000 |
|  |  |  | ω: | 0.04671 | 1.00000 | 31.53626 |  |  |
| M7 | 39 | -8790.104887 | p=0.23109 |  | q=2.29160 |  |  |  |
| M8 | 41 | -8789.346115 | p0=0.98990 | p=0.25249 | q=2.84344 |  | M7vs.M8 | 0.468251376 |
|  |  |  | (p1=0.01010) | ω=1.00000 |  |  |  |  |

ND4L

| Model | Np | LnL | Estemates of parameters | |  |  | Model  compared | LRT P-value |
| --- | --- | --- | --- | --- | --- | --- | --- | --- |
| M0 | 38 | -1855.079169 | ω: | 0.07856 |  |  |  |  |
| M1a | 39 | -1843.623558 | p: | 0.95948 | 0.04052 |  | M0vs.M1a | 0.000001697 |
|  |  |  | ω: | 0.06082 | 1.00000 |  |  |  |
| M2a | 41 | -1843.623558 | p: | 0.95948 | 0.02176 | 0.01876 | M1avs.M2a | 1.000000000 |
|  |  |  | ω: | 0.06082 | 1.00000 | 1.00000 |  |  |
| M7 | 39 | -1834.969997 | p=0.36748 |  | q=3.86662 |  |  |  |
| M8 | 41 | -1834.970977 | p0=0.99999 | p=0.36748 | q=3.86662 |  | M7vs.M8 | 0.999000500 |
|  |  |  | (p1=0.00001) | ω=5.34120 |  |  |  |  |

ND5

| Model | Np | LnL | Estemates of parameters | |  |  | Model  compared | LRT P-value |
| --- | --- | --- | --- | --- | --- | --- | --- | --- |
| M0 | 38 | -13085.010774 | ω: | 0.09609 |  |  |  |  |
| M1a | 39 | -12817.178453 | p: | 0.87716 | 0.12284 |  | M0vs.M1a | 0.000000000 |
|  |  |  | ω: | 0.04859 | 1.00000 |  |  |  |
| M2a | 41 | -12817.178453 | p: | 0.87716 | 0.05919 | 0.06364 | M1avs.M2a | 1.000000000 |
|  |  |  | ω: | 0.04859 | 1.00000 | 1.00000 |  |  |
| M7 | 39 | -12712.509597 | p=0.21869 |  | q=1.57587 |  |  |  |
| M8 | 41 | -12705.955329 | p0=0.97022 | p=0.26854 | q=2.69669 |  | M7vs.M8 | 0.001424051 |
|  |  |  | (p1=0.02978) | ω=1.08336 |  |  |  |  |

ND6

| Model | Np | LnL | Estemates of parameters | |  |  | Model  compared | LRT P-value |
| --- | --- | --- | --- | --- | --- | --- | --- | --- |
| M0 | 38 | -3508.371731 | ω: | 0.11101 |  |  |  |  |
| M1a | 39 | -3499.443793 | p: | 0.92810 | 0.07190 |  | M0vs.M1a | 0.000023828 |
|  |  |  | ω: | 0.09507 | 1.00000 |  |  |  |
| M2a | 41 | -3499.443793 | p: | 0.92810 | 0.03340 | 0.03850 | M1avs.M2a | 1.000000000 |
|  |  |  | ω: | 0.09507 | 1.00000 | 1.00000 |  |  |
| M7 | 39 | -3474.826951 | p=0.67355 |  | q=4.84316 |  |  |  |
| M8 | 41 | -3474.827287 | p0=0.99999 | p=0.67358 | q=4.84355 |  | M7vs.M8 | 0.999650061 |
|  |  |  | (p1=0.00001) | ω=1.00000 |  |  |  |  |
