# Supplementary material for: Complete Mitochondrial Genome Sequencing of Asian Glass Lizards (Anguidae: Dopasia): Comparative Analysis With Limbless Anguids and New Insights Into the Adaptive Evolution of Protein‐Coding Genes
Source: Ecol Evol. 2025 Dec 25;15(12):e72811. doi: 10.1002/ece3.72811 (PMC12740153; doi:10.1002/ece3.72811)
Supplement: Supplementary file 8 — Table S4: Candidate codon sites under positive selection from three Datamonkey tests. [file ECE3-15-e72811-s005.docx]

TABLE S4. Candidate codon sites under positive selection from three Datamonkey tests.

|  | MEME |  |  |  | FUBAR |  |  | SLAC |  |
| --- | --- | --- | --- | --- | --- | --- | --- | --- | --- |
| Gene | Codon | P-value |  |  | Codon | Prob. |  | Codon | *P*-value |
| ATP6 | 219 | 0.02 |  |  |  |  |  |  |  |
| ATP8 | 48 | 0.02 |  |  | 7 | >0.9 |  |  |  |
| COXⅠ | 42 | 0.05 |  |  |  |  |  |  |  |
| COXⅡ | 132 | 0.03 |  |  |  |  |  |  |  |
|  | 155 | 0.05 |  |  |  |  |  |  |  |
| COXⅢ | 23 | 0.01 |  |  |  |  |  |  |  |
|  | 33 | 0.05 |  |  |  |  |  |  |  |
|  | 41 | 0.01 |  |  | 41 | >0.9 |  | 41 | 0.01 |
|  | 151 | 0.05 |  |  |  |  |  |  |  |
| Cyt *b* | 3 | 0.02 |  |  | 3 | >0.9 |  | 3 | 0.02 |
|  |  |  |  |  | 239 | >0.9 |  | 239 | 0.04 |
| ND1 | 2 | 0.01 |  |  | 2 | >0.9 |  |  |  |
| ND2 | 210 | 0.05 |  |  | 274 | >0.9 |  | 274 | 0.04 |
| ND3 | 2 | 0.03 |  |  |  |  |  |  |  |
| ND4 | 21 | 0.01 |  |  | 21 | >0.9 |  |  |  |
|  | 26 | 0.05 |  |  | 26 | >0.9 |  | 26 | 0.04 |
|  | 188 | 0.05 |  |  | 182 | >0.9 |  |  |  |
|  | 260 | 0.05 |  |  |  |  |  |  |  |
|  | 404 | 0.03 |  |  |  |  |  |  |  |
|  | 422 | 0.03 |  |  |  |  |  |  |  |
| ND5 | 33 | 0.03 |  |  |  |  |  |  |  |
|  | 108 | 0.05 |  |  |  |  |  |  |  |
|  | 337 | 0.04 |  |  |  |  |  |  |  |
|  | 470 | 0.03 |  |  |  |  |  |  |  |
|  | 487 | 0.04 |  |  |  |  |  |  |  |
|  | 499 | 0.05 |  |  |  |  |  |  |  |
|  | 590 | 0.01 |  |  |  |  |  |  |  |
|  | 595 | 0.00 |  |  |  |  |  |  |  |
|  | 597 | 0.00 |  |  |  |  |  |  |  |
|  | 605 | 0.01 |  |  | 605 | >0.9 |  |  |  |
|  | 607 | 0.01 |  |  | 607 | >0.9 |  | 607 | 0.02 |
| ND6 | 100 | 0.01 |  |  | 103 | >0.9 |  |  |  |
|  | 148 | 0.02 |  |  | 105 | >0.9 |  |  |  |
