# Supplementary material for: Complete Mitochondrial Genome Sequencing of Asian Glass Lizards (Anguidae: Dopasia): Comparative Analysis With Limbless Anguids and New Insights Into the Adaptive Evolution of Protein‐Coding Genes
Source: Ecol Evol. 2025 Dec 25;15(12):e72811. doi: 10.1002/ece3.72811 (PMC12740153; doi:10.1002/ece3.72811)
Supplement: Supplementary file 9 — Table S5: Neutral and deleterious mutation sites predicted in PROVEAN. [file ECE3-15-e72811-s007.docx]

Table S5. Neutral and deleterious mutation sites predicted in PROVEAN.

| Mitogenome position | | Mutation effect analysis- PROVEAN | |  |
| --- | --- | --- | --- | --- |
| Gene | Position | Prediction(cutoff=-2.5) | Score | Variant |
| ATP6 | 219 | Neutral |  |  |
| ATP8 | 7 | Neutral |  |  |
|  | 48 | Deleterious | -3.194 | N↔C |
| COXⅠ | 42 | Neutral |  |  |
| COXⅡ | 132 | Neutral |  |  |
|  | 155 | Neutral |  |  |
| COXⅢ | 23 | Neutral |  |  |
|  | 33 | Neutral |  |  |
|  | 41 | Neutral |  |  |
|  | 151 | Neutral |  |  |
| CYTB | 3 | Neutral |  |  |
|  | 239 | Neutral |  |  |
| ND1 | 2 | Neutral |  |  |
| ND2 | 210 | Neutral |  |  |
|  | 274 | Neutral |  |  |
| ND3 | 2 | Neutral |  |  |
| ND4 | 21 | Neutral |  |  |
|  | 26 | Neutral |  |  |
|  | 182 | Neutral |  |  |
|  | 188 | Neutral |  |  |
|  | 260 | Deleterious | -7.916 | Y↔C |
|  |  | Deleterious | -4.479 | Y↔H |
|  |  | Deleterious | -3.345 | C↔H |
|  | 404 | Neutral |  |  |
|  | 422 | Neutral |  |  |
| ND5 | 33 | Neutral |  |  |
|  | 108 | Neutral |  |  |
|  | 337 | Deleterious | -7.442 | F↔S |

|  | 470 | Deleterious | -3.085 | I↔T |
| --- | --- | --- | --- | --- |
|  | 487 | Neutral |  |  |
|  | 499 | Neutral |  |  |
|  | 590 | Neutral |  |  |
|  | 595 | Neutral |  |  |
|  | 597 | Neutral |  |  |
|  | 605 | Neutral |  |  |
|  | 607 | Neutral |  |  |
| ND6 | 100 | Neutral |  |  |
|  | 103 | Deleterious | -3.512 | G↔M |
|  |  | Deleterious | -2.886 | G↔E |
|  |  | Deleterious | -3.896 | G↔L |
|  | 105 | Deleterious | -4.640 | W↔G |
|  |  | Deleterious | -6.868 | W↔C |
|  |  | Deleterious | -6.857 | W↔L |
|  |  | Deleterious | -2.921 | G↔L |
|  |  | Deleterious | -3.329 | G↔C |
|  | 148 | Deleterious | -4.560 | Y↔L |
