# Supplementary material for: Complete Mitochondrial Genome Sequencing of Asian Glass Lizards (Anguidae: Dopasia): Comparative Analysis With Limbless Anguids and New Insights Into the Adaptive Evolution of Protein‐Coding Genes
Source: Ecol Evol. 2025 Dec 25;15(12):e72811. doi: 10.1002/ece3.72811 (PMC12740153; doi:10.1002/ece3.72811)
Supplement: Supplementary file 10 — Table S6: Amino acid composition of positive selection sites (Datamonkey). [file ECE3-15-e72811-s002.docx]

TABLE S6. Amino acid composition of positive selection sites (Datamonkey).

| Taxon | Accession number | ATP6 | ATP8 | | COXⅠ | COXⅡ | |  | COXⅢ | | | |  | Cyt *b* | | ND1 | ND2 | | ND3 |  |
| --- | --- | --- | --- | --- | --- | --- | --- | --- | --- | --- | --- | --- | --- | --- | --- | --- | --- | --- | --- | --- |
|  |  |  |  |  |  |  |  |  |  |  |  |  |  |  |  |  |  |  |  |  |
|  | | 219 | 7 | 48 | 42 | 132 | 155 |  | 23 | 33 | 41 | 151 |  | 3 | 239 | 2 | 210 | 274 | 2 |  |
| *Dopasia gracilis* B5008 |  | I | A | S | S | F | S |  | A | M | N | L |  | T | A | T | L | A | T |  |
| *Dopasia gracilis* MTX |  | I | A | S | S | F | S |  | A | M | N | L |  | T | A | T | L | A | T |  |
| *Dopasia gracilis* G1942 |  | I | A | N | S | F | S |  | A | M | R | L |  | T | A | T | L | T | T |  |
| *Dopasia gracilis* S0867 |  | I | A | N | S | F | S |  | A | M | R | L |  | T | A | T | L | T | T |  |
| *Dopasia gracilis* GZ18001 |  | I | A | N | S | F | S |  | A | M | R | L |  | T | A | T | L | T | T |  |
| *Dopasia gracilis* GD04 |  | L | S | N | S | F | S |  | A | M | N | I |  | M | A | T | L | A | V |  |
| *Dopasia harti* TW |  | L | A | N | S | F | S |  | A | M | S | L |  | L | A | T | L | A | V |  |
| *Dopasia harti* CB |  | L | S | N | S | F | S |  | A | M | S | L |  | L | T | T | L | A | V |  |
| *Dopasia hainanensis* | MN640999 | L | S | N | S | F | S |  | A | M | S | L |  | L | T | T | L | A | V |  |
| *Dopasia harti* | KF806482 | L | S | N | S | F | S |  | A | M | S | I |  | M | A | T | L | A | V |  |
| *Dopasia harti* | KF279681 | L | S | N | S | F | S |  | A | M | S | I |  | P | A | T | L | A | V |  |
| *Dopasia gracilis* | MN661343 | I | A | N | G | F | S |  | A | M | R | L |  | T | A | T | L | T | T |  |
| *Dopasia gracilis* | KJ941042 | I | A | N | S | F | S |  | A | M | R | L |  | T | A | A | L | T | T |  |
| *Dopasia gracilis* | KU885977 | I | A | S | S | F | S |  | A | M | N | L |  | T | A | T | L | A | V |  |
| *Dopasia sokolovi* | OP493527 | I | N | N | S | F | S |  | A | M | N | L |  | P | T | T | L | T | T |  |
| *Anguis graeca* | OP493517 | L | G | N | S | F | S |  | A | M | A | I |  | T | A | A | L | T | V |  |
| *Anguis graeca* | KX236331 | L | G | N | S | F | S |  | A | M | A | I |  | T | A | A | L | A | V |  |
| *Anguis fragilis* | OP493519 | L | N | S | S | F | T |  | A | M | I | I |  | P | A | A | L | A | V |  |
| *Anguis fragilis* | EU443256 | L | N | S | S | F | T |  | A | M | I | I |  | P | A | A | L | A | V |  |
| *Anguis fragilis* | OP493518 | L | N | S | S | F | S |  | A | M | T | I |  | P | A | A | L | A | V |  |
| *Anguis fragilis* | MN122840 | L | N | S | S | F | S |  | A | M | T | I |  | P | A | A | L | A | V |  |
| *Anguis colchica* | OP493520 | L | S | D | S | F | S |  | A | M | A | I |  | T | A | A | L | T | V |  |
| *Anguis colchica* | OP493521 | L | S | D | S | F | S |  | A | M | A | I |  | T | A | A | L | T | V |  |
| *Anguis colchica* | OP493522 | L | S | S | S | F | S |  | A | L | T | I |  | M | T | A | L | T | V |  |
| *Anguis colchica* | OP493523 | L | S | G | S | F | S |  | A | L | I | I |  | T | A | A | L | T | M |  |
| *Anguis colchica* | OP493529 | L | S | S | S | F | S |  | A | L | T | I |  | T | A | A | L | A | V |  |
| *Anguis colchica* | KX236330 | L | S | D | S | F | S |  | A | M | A | I |  | T | A | A | L | T | V |  |
| *Anguis veronensis* | OP493530 | L | S | N | S | F | S |  | A | M | V | I |  | A | A | A | L | A | V |  |
| *Anguis veronensis* | KX236332 | L | S | N | S | F | S |  | A | M | V | I |  | T | A | A | L | A | V |  |
| *Anguis cephallonica* | OP493528 | L | N | N | S | F | S |  | A | M | T | I |  | T | A | A | L | T | V |  |
| *Anguis cephallonica* | KU052866 | L | N | N | S | F | S |  | A | M | T | I |  | T | V | A | L | A | V |  |
| *Pseudopus apodus* | OP493524 | L | N | N | S | F | S |  | A | M | A | I |  | L | A | T | T | T | V |  |
| *Pseudopus apodus* | OP493526 | L | N | N | S | F | S |  | A | M | A | I |  | L | A | A | T | A | V |  |
| *Pseudopus apodus* | OP493525 | L | S | N | S | F | S |  | A | M | A | I |  | L | A | A | T | T | V |  |
| *Ophisaurus attenuatus* | EU747729 | L | S | C | S | H | S |  | Q | M | A | I |  | L | M | H | L | A | S |  |
| *Abronia graminea* | AB080273 | L | G | N | S | F | S |  | A | M | T | I |  | A | M | H | L | V | T |  |

|  |  |  | | | | | | |  |  | | | | | | | | | | |  |  | | | |  |
| --- | --- | --- | --- | --- | --- | --- | --- | --- | --- | --- | --- | --- | --- | --- | --- | --- | --- | --- | --- | --- | --- | --- | --- | --- | --- | --- |
| Taxon | Accession number | ND4 | | | | | | |  | ND5 | | | | | | | | | | |  | ND6 | | | |  |
|  |  |  |  |  |  |  |  |  |  |  |  |  |  |  |  |  |  |  |  |  |  |  |  |  |  |  |
|  | | 21 | 26 | 182 | 188 | 260 | 404 | 422 |  | 33 | 108 | 337 | 470 | 487 | 499 | 590 | 595 | 597 | 605 | 607 |  | 100 | 103 | 105 | 148 |  |
| *Dopasia gracilis* B5008 |  | R | M | S | Q | Y | T | T |  | G | H | F | F | A | T | S | T | I | A | T |  | V | L | L | L |  |
| *Dopasia gracilis* MTX |  | R | M | S | Q | Y | T | T |  | G | H | F | F | A | T | S | T | I | A | T |  | V | L | L | L |  |
| *Dopasia gracilis* G1942 |  | R | M | S | Q | Y | T | T |  | G | H | F | L | T | M | S | A | I | T | P |  | V | L | L | L |  |
| *Dopasia gracilis* S0867 |  | R | M | S | Q | Y | T | T |  | G | H | F | L | T | M | S | A | I | T | P |  | V | L | L | L |  |
| *Dopasia gracilis* GZ18001 |  | R | M | S | Q | Y | T | T |  | G | H | F | L | T | M | S | A | I | T | P |  | V | L | L | L |  |
| *Dopasia gracilis* GD04 |  | R | T | S | Q | Y | T | T |  | K | H | F | V | A | T | S | A | I | T | S |  | V | L | W | L |  |
| *Dopasia harti* TW |  | R | S | S | Q | Y | T | T |  | K | H | F | V | T | T | S | I | I | T | P |  | V | L | L | L |  |
| *Dopasia harti* CB |  | R | S | F | Q | Y | A | T |  | K | H | F | L | T | T | S | T | I | T | P |  | V | L | C | L |  |
| *Dopasia hainanensis* | MN640999 | R | S | F | Q | Y | A | T |  | K | H | F | L | T | T | S | T | I | T | P |  | V | L | C | L |  |
| *Dopasia harti* | KF806482 | R | S | S | Q | Y | T | T |  | K | H | F | V | A | T | S | A | I | T | S |  | V | L | W | L |  |
| *Dopasia harti* | KF279681 | R | S | S | Q | Y | T | T |  | K | H | F | V | A | T | M | L | T | A | F |  | V | W | W | Y |  |
| *Dopasia gracilis* | MN661343 | R | T | S | Q | Y | T | T |  | G | H | F | L | T | M | S | A | I | T | P |  | V | L | L | L |  |
| *Dopasia gracilis* | KJ941042 | R | M | S | Q | Y | T | T |  | G | H | F | L | T | M | S | T | I | T | P |  | V | L | L | L |  |
| *Dopasia gracilis* | KU885977 | R | M | S | Q | C | T | T |  | G | H | F | F | A | T | S | L | T | A | F |  | V | L | L | L |  |
| *Dopasia sokolovi* | OP493527 | G | T | S | Q | Y | T | T |  | K | H | F | T | T | T | S | S | I | T | P |  | V | L | L | L |  |
| *Anguis graeca* | OP493517 | S | A | A | Q | Y | T | T |  | K | S | F | I | T | T | T | L | I | T | F |  | L | G | W | L |  |
| *Anguis graeca* | KX236331 | P | A | T | Q | Y | A | T |  | K | S | F | I | M | T | T | L | I | T | F |  | G | G | W | L |  |
| *Anguis fragilis* | OP493519 | S | A | T | Q | Y | A | T |  | K | S | F | I | T | T | T | L | I | T | F |  | G | G | W | L |  |
| *Anguis fragilis* | EU443256 | S | A | T | Q | Y | A | T |  | K | S | S | I | T | T | T | L | I | T | F |  | G | G | W | L |  |
| *Anguis fragilis* | OP493518 | S | A | T | Q | Y | T | T |  | K | S | F | I | T | T | T | L | I | T | F |  | G | G | W | L |  |
| *Anguis fragilis* | MN122840 | S | A | T | Q | Y | T | T |  | K | S | F | I | T | T | T | L | I | T | F |  | G | G | W | L |  |
| *Anguis colchica* | OP493520 | S | A | A | Q | Y | A | T |  | K | S | F | I | M | V | T | L | I | T | F |  | G | E | W | L |  |
| *Anguis colchica* | OP493521 | S | A | A | Q | Y | A | T |  | K | S | F | I | M | V | T | L | I | T | F |  | G | G | W | L |  |
| *Anguis colchica* | OP493522 | S | T | A | Q | Y | A | T |  | K | S | F | I | M | V | T | L | F | A | F |  | G | G | W | L |  |
| *Anguis colchica* | OP493523 | S | T | A | Q | Y | A | T |  | K | S | F | V | M | V | T | L | I | T | F |  | G | G | W | L |  |
| *Anguis colchica* | OP493529 | S | A | A | Q | Y | A | T |  | K | S | F | V | M | I | T | L | I | T | F |  | G | G | W | L |  |
| *Anguis colchica* | KX236330 | S | A | A | Q | Y | A | T |  | K | S | F | I | M | V | T | L | I | T | F |  | G | G | W | L |  |
| *Anguis veronensis* | OP493530 | S | A | A | Q | Y | T | T |  | K | S | F | I | T | M | T | L | I | T | F |  | V | G | W | L |  |
| *Anguis veronensis* | KX236332 | S | A | A | Q | Y | T | T |  | K | S | F | I | T | M | T | L | I | T | F |  | V | G | W | L |  |
| *Anguis cephallonica* | OP493528 | P | A | V | P | Y | T | T |  | K | S | F | I | V | T | T | L | I | T | F |  | V | G | W | L |  |
| *Anguis cephallonica* | KU052866 | P | A | V | L | Y | T | T |  | K | S | F | I | V | T | T | L | I | T | F |  | V | G | W | L |  |
| *Pseudopus apodus* | OP493524 | P | T | I | Q | Y | T | T |  | K | S | F | I | A | T | M | S | I | P | F |  | V | G | L | L |  |
| *Pseudopus apodus* | OP493526 | S | T | T | Q | Y | T | T |  | K | S | F | I | T | T | M | S | I | L | F |  | V | G | L | L |  |
| *Pseudopus apodus* | OP493525 | Q | T | T | Q | H | T | T |  | K | S | F | I | T | T | M | S | I | L | F |  | A | G | L | L |  |
| *Ophisaurus attenuatus* | EU747729 | S | S | S | Q | Y | T | N |  | K | S | F | I | L | A | T | A | I | T | A |  | V | G | W | L |  |
| *Abronia graminea* | AB080273 | K | A | M | Q | Y | T | T |  | K | S | F | I | T | V | T | T | L | Q | N |  | L | M | G | L |  |
