# Supplementary material for: Complete Mitochondrial Genome Sequencing of Asian Glass Lizards (Anguidae: Dopasia): Comparative Analysis With Limbless Anguids and New Insights Into the Adaptive Evolution of Protein‐Coding Genes
Source: Ecol Evol. 2025 Dec 25;15(12):e72811. doi: 10.1002/ece3.72811 (PMC12740153; doi:10.1002/ece3.72811)
Supplement: Supplementary file 1 — Figure S1: Mitochondrial genome map of Dopasia gracilis B5008. Genes on the light and heavy strands are shown inside and outside the circle, respectively, with arrows indicating transcription direction. tRNA genes are colored blue and labeled with three‐letter amino acid codes. Maps for other individuals analyzed are similar. [file ECE3-15-e72811-s006.pdf]

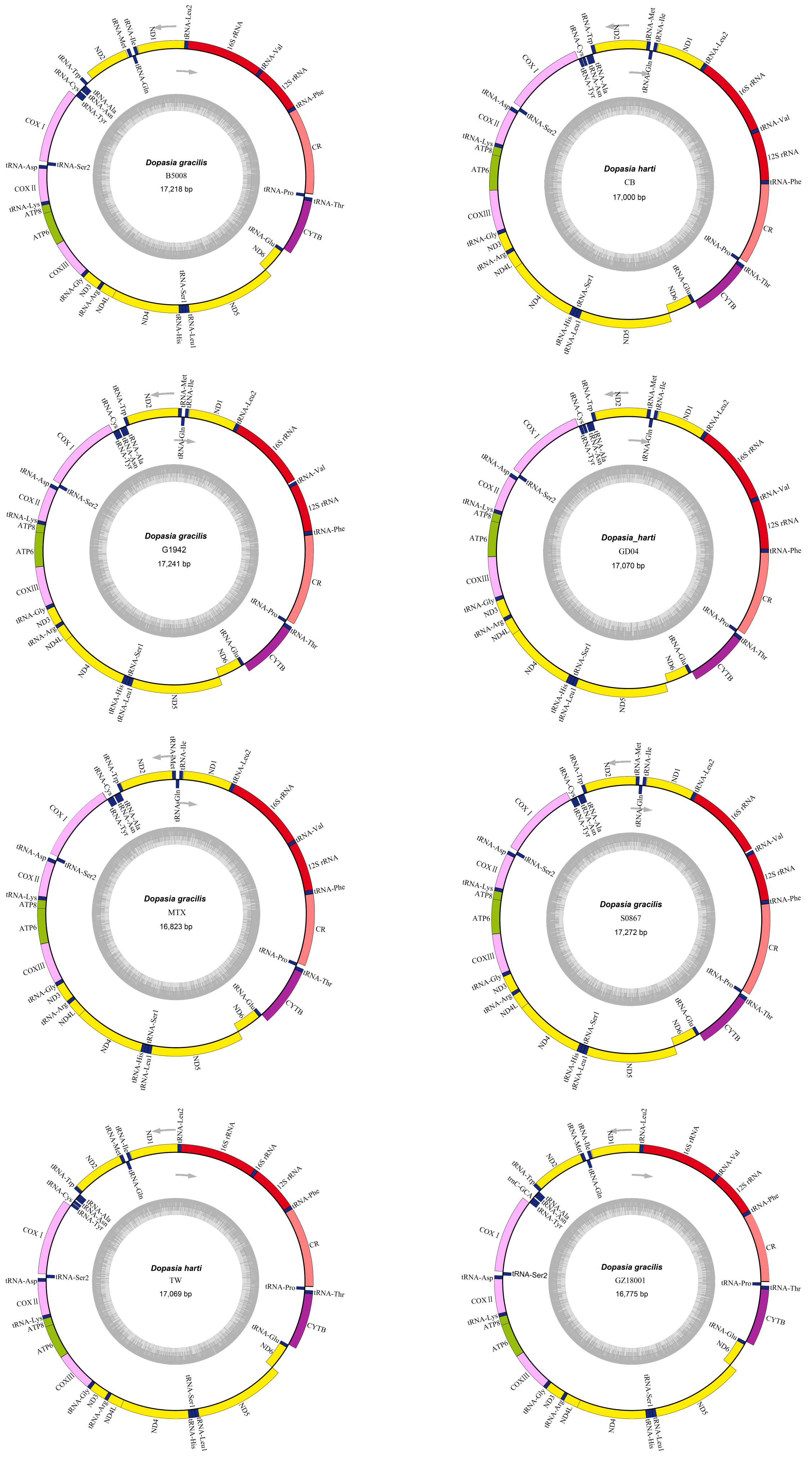

Dopasia gracilis
B5008
17,218 bp
Dopasia harti
CB
17,000 bp
Dopasia gracilis
G1942
17,241 bp
Dopasia_harti
GD04
17,070 bp
Dopasia gracilis
MTX
16,823 bp
Dopasia gracilis
S0867
17,272 bp
Dopasia harti
TW
17,069 bp
Dopasia gracilis
GZ18001
16,775 bp
